# KDAC6 alters cell morphology and motility through positive and negative modulation of F‐actin distribution

**DOI:** 10.1002/2211-5463.70302

**Published:** 2026-09-25

**Authors:** Taylor V. Joseph, Andrea B. Pham, Kiara E. Bornes, Razan H. Hammad, Chelsea A. Rousseve‐Ross, Thomas M. Huckaba, Terry J. Watt, Tasha B. Toro

**Affiliations:** ^1^ Department of Chemistry Xavier University of Louisiana New Orleans LA USA; ^2^ Department of Biology Xavier University of Louisiana New Orleans LA USA

**Keywords:** acetylation, actin, cell morphology, cell motility, histone deacetylase 6, RNA‐seq

## Abstract

Lysine deacetylase 6 (KDAC6 or HDAC6) has been associated with cell motility and deacetylation of cytoskeleton‐related proteins. KDAC6 is the only human KDAC having three identified major domains, including two catalytic domains (CD). Defining the roles of KDAC6 in cell motility has been constrained by limited understanding of domain‐specific contributions. Live and fixed cell imaging was used to characterize the effects of KDAC6 on F‐actin distribution, cell morphology, and cell motility of genetically modified HT1080 cells containing KDAC6 with an inactivated CD or knockout. CD1 inactivation resulted in cells that migrated faster and had more concentrated cortical F‐actin at the leading edge. Conversely, inactivation of CD2 resulted in slower migration, depleted cortical F‐actin, more stress fibers with poor alignment, and larger, flatter cells. Loss of both CD1 and CD2 activity in the knockout cell line resulted in phenotypes that were most similar to wild‐type cells. Hyperacetylation of α‐tubulin alone was insufficient to explain the observed phenotypes of the cell line with inactive CD2, as the knockout cell line had similar hyperacetylation but lacked most of the phenotypic changes. Altered gene expression implicated actin‐associated proteins as mediators of phenotypic effects. Although the cell lines exhibited variation in localization of putative KDAC6 targets cortactin and HSP90, these variations did not correlate with the observed phenotypes. Inactivation of CD2 resulted in more focal adhesions formed throughout cells, correlating with more stress fibers and reduced motility. Collectively, these results establish that each CD of KDAC6 has a distinctive and opposing role in F‐actin regulation.

## Graphical abstract

KDAC6 has been associated with cell motility and actin structures, but specific domain contributions are not established. The two catalytic domains have differential effects on motility and F‐actin regulation, affecting cortical F‐actin density, motility, stress fibers, and cell spreading. KDAC6 has a dual role in regulating F‐actin with significant effects on cell morphology and motility.

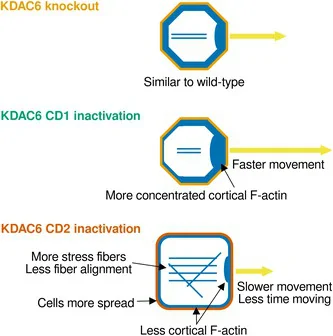

**Abbreviations**
6CD1m: HT1080 expressing KDAC6 with inactive CD1 (HT1080 KDAC8H216A)
6CD2m: HT1080 expressing KDAC6 with inactive CD2 (HT1080 KDAC8H611A)
6KO: HT1080 KDAC6 knockout
ATAT1: alpha‐tubulin N‐acetyltransferase 1 (UniprotKB Q5SQI0)
CD: catalytic domain
CTTN: Src substrate cortactin (UniprotKB Q14247)
HSP90: heat shock protein 90 α/β (UniprotKB P07900/P08238)
KDAC6: lysine deacetylase 6 (UniprotKB Q9UBN7)
n.s.: not significant
PBS: phosphate‐buffered saline
VCL: vinculin (UniprotKB P18206)
WT: wild‐type

Most animal cells have the ability to migrate, which is important for many normal physiological functions, such as developmental processes and wound healing [1]. The cytoskeleton is made up of actin filaments (F‐actin), microtubules, and intermediate filaments. Together, these structures facilitate movement of a cell through its environment in response to extracellular environmental cues that are transmitted through signal transduction pathways [2, 3]. Many cell types, including fibroblasts, typically rely on mesenchymal migration for movement. In this mode, cells first protrude at one end, form focal adhesions at their leading edge to anchor cells to the surface, and then contract at the opposite end [4]. These processes are the result of dynamic F‐actin assembly and disassembly into various structures, regulated by many actin‐binding proteins that facilitate the remodeling of the actin cytoskeleton [5]. Some cells instead move primarily in an amoeboid fashion, allowing them to traverse through existing matrix pores or vessels [6]. This motility mode relies on blebbing instead of adhesion for movement and allows faster movement of cells [2, 7, 8]. Some cells, including metastatic cancer cells, are capable of both types of movement, allowing migration plasticity to accommodate different environments [9, 10, 11]. Ultimately, both types of cell movement are facilitated by actin remodeling, which is highly regulated through signaling pathways and actin‐associated proteins [2]. Thus, aberrant expression and behavior of actin and actin‐associated proteins can lead to enhanced proliferation and motility, which promotes several disease states including cancer [12, 13].

During migration, actin remodeling typically results in a concentration of cortical actin at the leading edge of the migrating cells, often in the form of lamellipodia and/or membrane ruffles. These structures contain fast growing branched actin with barbed ends that push toward the cell membrane and appear as a mesh network under high‐resolution microscopy [3, 14]. Transient focal adhesions are also often present in these structures, functioning as a link between the actin cytoskeleton and the extracellular matrix, which provides mechanical force for migration and allows cells to receive external signals [15]. Stress fibers are another common actin structure, which contribute to both cell adhesion and cell motility through a variety of mechanisms. Stress fibers consist of antiparallel actin filaments that contain myosin and can provide contractile forces for the cell. Long ventral stress fibers form along the bottom of a cell and both ends attach to focal adhesions that are bound to the extracellular matrix or surface [1, 16, 17]. Stress fibers are typically oriented parallel to one another along the long axis of the cell, particularly in migrating cells [1, 5, 18].

Cytoskeletal remodeling and cell motility have been extensively studied for many decades; however, how these processes are regulated is still not well‐understood. Lysine deacetylase 6 (KDAC6 or HDAC6) is one regulatory protein that has been directly and indirectly associated with cell motility by multiple groups [19]. KDAC6 is a member of the lysine deacetylase enzyme family that is found in the cytosol and is the only member of the KDAC family known to contain two catalytic domains, designated CD1 and CD2 [20, 21]. While KDAC6 CD2 is capable of deacetylating a wide range of substrates *in vitro*, including α‐tubulin, CD1 appears capable of deacetylating only substrates with a C‐terminal acetyl‐lysine *in vitro* [22]. The narrower substrate specificity of CD1 compared to CD2 can be explained using observed differences in crystal structures of the active sites; however, the *in vivo* function of this domain remains unclear [23]. In addition to the two catalytic domains, KDAC6 also contains a zinc‐finger/ubiquitin binding domain that is involved in functions unrelated to catalysis, particularly transport of protein aggregates by simultaneous binding to ubiquitinated aggregates and dynein motors on microtubules [21, 24]. Aberrant expression or dysfunction of KDAC6 has been associated with multiple cancer types, leading to interest in KDAC6 as a therapeutic target [25, 26, 27, 28].

Previous studies have found that overexpression of wild‐type KDAC6 leads to increased cell motility, while loss of KDAC6 leads to decreased cell migration in transwell migration assays [20, 29, 30, 31]. It is not clear whether this role is deacetylation‐dependent, as overexpression of catalytically inactive mutants has not produced consistent results [20, 29, 30]. For example, experiments using NIH3T3 cells and MEFs, both mouse fibroblast lines, demonstrated that the increased motility observed upon overexpression of KDAC6 required the catalytic function [20, 29]. Conversely, another group reported that the catalytic function of KDAC6 was dispensable for enhanced motility upon overexpression in lymphocytes [30]. In contrast to these results, at least one report found that knockdown of KDAC6 had no effect on cell migration in a human fibroblast cell line [31]. It is therefore plausible that differences in cell type may be contributing to the observed differences in phenotype.

Several connections between KDAC6 and cell motility have been observed, and models have been proposed to explain the mechanism by which KDAC6 modulates cell motility. KDAC6 plays a role in modulating microtubule remodeling by deacetylating lysine 40 (K40) of α‐tubulin, which is the most well‐characterized KDAC6 substrate, having been validated both *in vivo* and *in vitro* [32, 33, 34]. Acetylation of α‐tubulin has been linked to cell migration through cross‐talk with the actin cytoskeleton mediated through myosin‐binding proteins and focal adhesions, although the evidence is mostly through manipulation of the α‐tubulin acetyltransferase ATAT1 [35, 36, 37, 38, 39]. Other acetylated proteins interact or co‐localize with actin and play a role in cell motility, providing a potential additional explanation for the effect of KDAC6 in cell movement [20, 29, 40, 41]. For example, cortactin (CTTN) and heat shock protein 90 (HSP90) are acetylated, involved in cell motility, and have both been linked to KDAC6; however, it is not clear whether they are direct targets of KDAC6 or whether acetylation is regulating their effect on cell movement [29, 40, 41, 42, 43, 44, 45, 46, 47].

Elucidating the roles of the KDAC6 catalytic domains on cellular processes has been challenging due to technical limitations, in particular the use of nonspecific inhibitors as well as the confounding influence of the third (ubiquitin‐binding) domain in experiments in which KDAC6 has been overexpressed or knocked down [34, 48, 49, 50]. To circumvent these issues and isolate the catalytic roles of KDAC6 CD1 and CD2, we developed two HT1080‐derivative cell lines in which KDAC6 has been mutated at the endogenous locus to inactivate a single catalytic domain [51]. These lines retain endogenous expression of KDAC6, but a single catalytic domain of the protein is genetically inactivated in each line, resulting in a single amino acid change. Analysis of gene expression data comparing these cell lines revealed that inactivation of CD1 and CD2 resulted in distinct changes in gene expression, suggesting that each catalytic domain of KDAC6 has distinct functions in cells. Interestingly, clusters of genes related to gene ontology (GO) processes involved in cell motility and cytoskeleton organization were significantly downregulated in cells in which KDAC6 CD2 (but not CD1) was genetically inactivated [51]. HT1080 cells were isolated from a fibrosarcoma and are known to be highly motile, making them an ideal system to study effects on cell motility [52, 53, 54]. Here, we used these cell lines [6CD1m (HT1080 KDAC6H216A) and 6CD2m (HT1080 KDAC6H611A)], along with a newly generated KDAC6 knockout line (6KO) in the same genetic background, to characterize the contributions of each KDAC6 catalytic domain on cell motility. We report that inactivation of each catalytic domain leads to distinct actin‐based cytoskeletal changes, resulting in opposing cell motility phenotypes.

## Materials and methods

### Cell growth and construction of the KDAC6 knockout cell line

HT1080 cells [HT1080 (HT1080) 9ATCC CCL‐121] were obtained from ATCC. 6CD1m and 6CD2m cell lines were generated from HT1080 cells using CRISPR/Cas9 during a previous study [51]. The 6KO cell line was generated using a similar protocol targeting the KDAC6H216 locus. For this line, pSpCas9(BB)‐2A‐Puro (PX459) V2.0 containing the 6H216‐specific gRNA sequence (CACCG AGCCA TCCAT AAGAC TGTGC) was transfected into HT1080 cells. Transformed cells were selected using 2.0 μg·mL^−1^ puromycin. Cells were then plated in 96‐well plates to achieve single colony dilutions. Candidates were genotyped by genomic DNA prep, PCR, and DNA sequencing of the PCR product to confirm the presence of a nonsense mutation using a protocol previously described in detail [51]. Four independent confirmed knockout isolates were pooled to create the 6KO line. All cell lines were cultured as previously described. Briefly, cells were cultured in growth media [minimal essential medium with Earle's balanced salts and L‐glutamine (MEM) supplemented with 10% fetal bovine serum, 50 U^−1^ Penicillin and 50 μg·mL^−1^ Streptomycin] and incubated at 37 °C with 5% CO_2_ [51].

### Immunoblotting

All immunoblots were performed as described previously, using approximately 30 μg lysate for each sample [51]. The following primary antibodies (from Cell Signaling Technologies, Danvers, MA, USA, unless otherwise noted) were used in this study at a 1 : 1000 dilution: α‐tubulin (3873), α‐tubulin‐acetyl‐K40 (5335), KDAC6 (5778), HSP90 (4877), Cortactin (3503), and actin (Developmental Studies Hybridoma Bank, Iowa City, IA JLA20). Appropriate secondary antibodies (Invitrogen 31464 or 31432; Thermo Fisher, Waltham, MA, USA) were used at 1 : 5000 and incubated for 1 h at room temperature and detected using either Supersignal West Pico Plus Chemiluminescence substrate (Thermo Fisher) or Supersignal West Femto Maximum Sensitivity Substrate (Thermo Fisher). Immunoblot images were processed uniformly with a linear intensity range and for the maximum intensity range without exceeding saturation or cropping signal in any bands.

### Gene expression analysis

RNA‐seq analysis and data processing for the 6KO line were performed as previously described for the 6CD1m and 6CD2m lines, except using a false discovery rate adjustment of *α* = 0.001 [51]. Gene Ontology analysis was performed as previously described, but using the basic ontology set 2025‐06‐01 and the GO human annotation set 2025‐06‐01 [51]. The raw gene expression data for the inactivated third domain cell line was downloaded and analyzed using the same conditions as for the 6KO line [55].

### Immunofluorescence and fluorescent microscopy

Cells were grown under standard conditions on plasma‐treated glass coverslips for at least 2 days until they reached 30–50% confluence. For fixation, formaldehyde was added to media to a 4% final concentration and incubated at room temperature for 15 min. Cells were washed in phosphate‐buffered saline (PBS) pH 7.4 and then submerged in ice‐cold ethanol for 10 min. After washing in PBS three times for 10 min each, cells were incubated in 5% goat serum in PBS at room temperature for 30 min. Coverslips were incubated with primary antibody for 2 h. The same primary antibodies used for immunoblotting were used in these experiments, with the addition of VCL (Invitrogen 700062; Thermo Fisher). All antibodies were used at a 1 : 50 dilution in 5% goat serum in PBS, unless otherwise noted. After washing in PBS three times for 10 min each, coverslips were incubated with secondary antibodies for 1 h. Alexa Fluor™ 488 and 568 secondary antibodies (Thermo Fisher, Waltham, MA USA) against the appropriate species were used at a 1 : 200 dilution in 5% goat serum in PBS. After washing in PBS, coverslips were mounted using SlowFade™ diamond antifade mountant with DAPI (Thermo Fisher). For actin visualization, cells were grown and fixed using the same method for immunofluorescence. After washing with phosphate‐buffered saline (PBS), cells were incubated with an Alexa Fluor™ 488 phalloidin probe (Thermo Fisher) at a 1 : 500 dilution in PBS containing 5% goat serum for 1 h at room temperature. Images were acquired and processed as previously described using a Nikon Eclipse Ti microscope with a 60X (1.40 NA) oil objective [50]. Excitation of fluorescence samples was performed using an Excelitas X‐Cite LED array using LEDs with peak excitation wavelengths of 365, 475, and 555 nm. Matching filter cubes in the Nikon Ti turret were: AT350/50×, ET460/50 m, T400lp; ET470/40×, ET525/45 m, T495lpxr; and ET545/25×, ET605/70 m, T565lpxr. All imaging was carried out with 100‐ or 200‐ms exposure time for each channel (kept consistent for each protein), with constant power to the LED array, and the gain set to zero on the EMCCD camera (iXon Ultra). Bias in cell images was minimized by capturing images in a contiguous array on each slide. The microscope was focused on the bottom plane of the visible cells prior to capturing each image, and only cells not apparently undergoing mitosis or cytokinesis (i.e., visually presenting as interphase) were used for analysis.

To determine the α‐tubulin ac‐K40/α‐tubulin ratio, a freehand line was drawn around the perimeter of each cell using the α‐tubulin signal, and the total grayscale value inside each cell in each the α‐tubulin and α‐tubulin ac‐K40 channels was measured.

To determine cell area, a freehand line was drawn around phalloidin‐stained images of individual isolated cells. The cell body was determined as all pixels with an intensity of at least 5% of the maximum intensity in each image, and all pixels below this threshold that were surrounded by pixels above the threshold (i.e., holes in the resulting space were filled in). The resulting selection of pixels was visually validated and then the number of pixels converted to an area of each cell.

To evaluate stress fiber count and alignment, a freehand line was drawn inside phalloidin‐stained images of individual isolated and nonisolated cells to exclude the cortex and ambiguous features arising from cell–cell contacts. The image was cropped to only the area inside of the freehand line, such that each image represented the noncortical region of a single cell. FilamentSensor 2.0 was used to identify actin stress fibers in the resulting images of individual cells from each cell line and to assign an angle to each fiber [56]. For cells with 4 or more stress fibers, the average angle of all stress fibers within a single cell was set to 0°. The deviation of each stress fiber from the mean of the corresponding cell was then calculated in the range −90° to +90°.

To evaluate the fraction of the total amount of a protein at the cortex, a variation of line analysis was used. A freehand line was drawn around phalloidin‐stained or immunofluorescence images of individual isolated cells to create a mask. Separately, a region of each image that lacked cells was marked by a separate freehand line as a background region. The resulting background region, phalloidin or immunofluorescence image, and a matching DAPI‐stained image were then processed using an automated line analysis algorithm implemented in Python. First, the nucleus of each cell was located by finding the largest contiguous block of pixels that were at least 40% of the maximum intensity within the masked region of the DAPI image, after background selection. Cells were excluded if the nuclear size was below a threshold suggesting that the cell was actively undergoing mitosis. Cells with multiple nuclei were retained in the analysis. Second, the largest contiguous region of most intense signal from phalloidin or the secondary antibody was identified using the brightest 3% of pixels within the masked region, after background selection. Third, the line through the center of the nucleus and the center of the region identified in the previous step was found, and extended across the entire masked region. Fourth, additional lines were calculated at successive angles of 5° to the original line and passing through the center of the nucleus, resulting in 36 lines spanning the masked region. Each line was one pixel wide. Fifth, the signal intensity along each line was analyzed using the find_peaks function in the signal module of SciPy [57] to find the most intense signal peak along each portion of the line from the nucleus center to the mask edge, with a minimum peak requirement of a width of 2 and a prominence of 50% of the maximum value along the line. Manual review determined that these peak settings consistently identified the cell cortex. Pixels within each peak were then assigned to the cortex. The resulting cortical and noncortical pixels from each line analysis were then pooled nonredundantly (i.e., pixels belonging to multiple lines within the nuclear region were only counted once, always as noncortical). Finally, the sum of the signal intensity of the pooled cortical pixels was divided by the sum of the signal intensity of all pixels (cortical and non‐cortical) to determine the fraction of signal corresponding to the cortex for a single cell.

The F‐actin density index was calculated using the same set of images as used for the analysis of the fraction of total F‐actin at the cortex. The process described for determining the fraction of signal at the cortex was followed through the third step of the algorithm above. Subsequently, two lines were found at angles of ±45° to the original line and through the center of the nucleus, defining a quadrant of the cell that included the most intense block of signal. The index was calculated as the mean of the signal intensity for the masked pixels within the quadrant divided by the mean of the signal intensity of the masked pixels outside the quadrant.

The cortical F‐actin polarization ratio was calculated, using the same images as the prior two analyses, by finding the line through the most intense region of cortical F‐actin and the center of the nucleus, extended through to the cortex on the far side of the nucleus from the most intense region. The algorithm used was the same as that for total cortical F‐actin analysis, but using only a single line with a line width of seven pixels, summing the cortical peak signal across the width of the line. For cells lacking a detected second peak due to low signal, the signal was defined as 1% of the intensity of the peak found in the most intense region and the same peak width. Each peak signal was averaged across the peak width to obtain an average cortical signal at each end of the cell. The cortical F‐actin polarization ratio was then found as the average value from the peak corresponding to the most intense cortical F‐actin region over the average signal from the peak on the opposing end of the cell.

Evaluation of organized interior vinculin was performed by visual analysis on each VCL image, independently by two researchers. Each cell was annotated as either having or not having organized VCL signal throughout a substantial portion of the cell interior and not just at the cell edge. Discrepancies in annotation were discussed to reconcile minor differences in individual classification thresholds and determine a final classification for those cells.

### Live‐cell imaging and motility assays

All live cell imaging was performed with either an Incucyte S3 or Incucyte SX5 live cell analysis instrument (Sartorius, Göttingen, Germany). For motility experiments, approximately 4000 cells per well in 200 μL growth media were plated in an Incucyte Imagelock 96‐well microplate (Sartorius). Phase contrast images at 10X magnification were taken in Imagelock mode every 5 min for 36 h. Time‐lapse images for each field were opened as a stack in imagej, and individual cells were tracked over 4 h from a variety of initial time points, using the MTrackJ plugin and a visual estimate of the cell center [58]. Artificial track noise was reduced by smoothing tracks using a Gaussian kernel smoother with the two nearest neighbor track points in time. Random noise caused by morphology changes leading to a shifting cell center but unrelated to net movement was reduced using an algorithm implemented in Python. First, for each track point, the prior track point closest in time that resulted in a net change of at least 20 μm was identified. Second, for each net movement, a distance value corresponding to the net distance divided by the number of intervening time points was assigned to all track points from the earlier point up to, but not including, the initial point. Third, after evaluating the entire track, all distance values assigned to a particular track point were averaged to obtain a final net distance change between track points. Finally, total distance was calculated as the sum of all these final net distance changes. The average velocity of moving cells was calculated by dividing the total distance by the number of track points with a nonzero net distance.

Bulk migration was determined by a scratch assay. Cells were plated at a density of 0.4–0.8 × 10^4^ cells per well in 200 μL growth media in an Incucyte Imagelock 96‐well microplate (Sartorius) and incubated under standard conditions. After 24 h, 100 μL growth media was removed and a scratch was made using the Incucyte Woundmaker Tool (Sartorius). Wells were washed twice with fresh media and then filled with 200 μL growth media per well. Cells were imaged at 10X magnification in Imagelock mode every 30 min for 24 h. Velocity of bulk migration was analyzed by using the first 4 h of images. The open area of the scratch was identified by contrast using a Python script, then the average distance between the two edges of the resulting area was calculated and divided by 30 min to obtain a velocity. The overall velocity was calculated as the average of the eight velocities between the frames.

For live cell F‐actin imaging, cells were plated in Incucyte Imagelock 96‐well plates as described above and incubated under standard conditions for 24 h. For each well, 0.2 μL Lipofectamine 2000 transfection reagent (Thermo Fisher Scientific, Waltham, MA) combined with 50 ng pEGCP‐C1 Lifeact‐EGFP (a gift from Dysche Mullins, Addgene plasmid #58470, Watertown, MA) was diluted into 12.5 μL MEM media and incubated at room temperature for 20 min [59, 60]. The mixture was added to 200 μL fresh MEM + 10% FBS and added to each well. Media were replaced after 4 h. After an additional 12 h, phase contrast and green fluorescent (300 ms) live cell images were acquired in standard mode at 5 min intervals at 20X magnification.

### Flow cytometry

Cells were grown to confluence under standard conditions. After trypsinization, cells were centrifuged at 500× ***g*** for 5 min at 4 °C. Cells were washed twice in ice‐cold PBS and then resuspended in 500 μL ice‐cold PBS. 4.5 mL ice‐cold 70% ethanol was added while vortexing. Cells were stored at 4 °C until further processing. Ethanol‐fixed cells were centrifuged at 500× ***g*** for 5 min, resuspended in 5 mL PBS, and incubated at room temperature for 10 min to equilibrate. Cells were then treated with 200 μg·mL^−1^ RNase A at 37 °C for 15 min protected from light and centrifuged at 500× ***g*** for 5 min. Cells were resuspended in 5 μg mL^−1^ propidium iodide, incubated at 37 °C for 30 min protected from light, and analyzed by flow cytometry using a FACSymphony A1 (BD Biosciences, Milpitas, CA, USA). Data were gated to eliminate double cells by front scatter height vs. area and then gated to eliminate debris by side scatter area vs. front scatter area. Relative cell volume was determined using the front scatter area of the resulting cell population.

### Statistical analysis

Most datasets were compared using a two‐sided Mann–Whitney *U* test to minimize effects of non‐normal distributions and outliers, and also evaluated for effect size using Cohen's *d*. Differences were considered significant if both *P* (adjusted) < 0.05 and Cohen's *d* magnitude > 0.2. The chi‐squared test was used to identify differences in stress fiber angles, with a bin size of 15°, for *P* (adjusted) < 0.05. The Fisher exact test was used to identify differences in the frequency of organized interior VCL, for *P* (adjusted) < 0.05. Significance values for all tests are reported as the adjusted p value after applying the Bonferroni correction for multiple testing of pairwise comparisons. Sample size, mean, standard deviation, median, test statistics, and unadjusted p values for every data set tested for significance are listed in Table S1.

## Results

### Gene expression changes in KDAC6 knockout are distinct from inactivation of CD1 or CD2

**Fig. 1:**
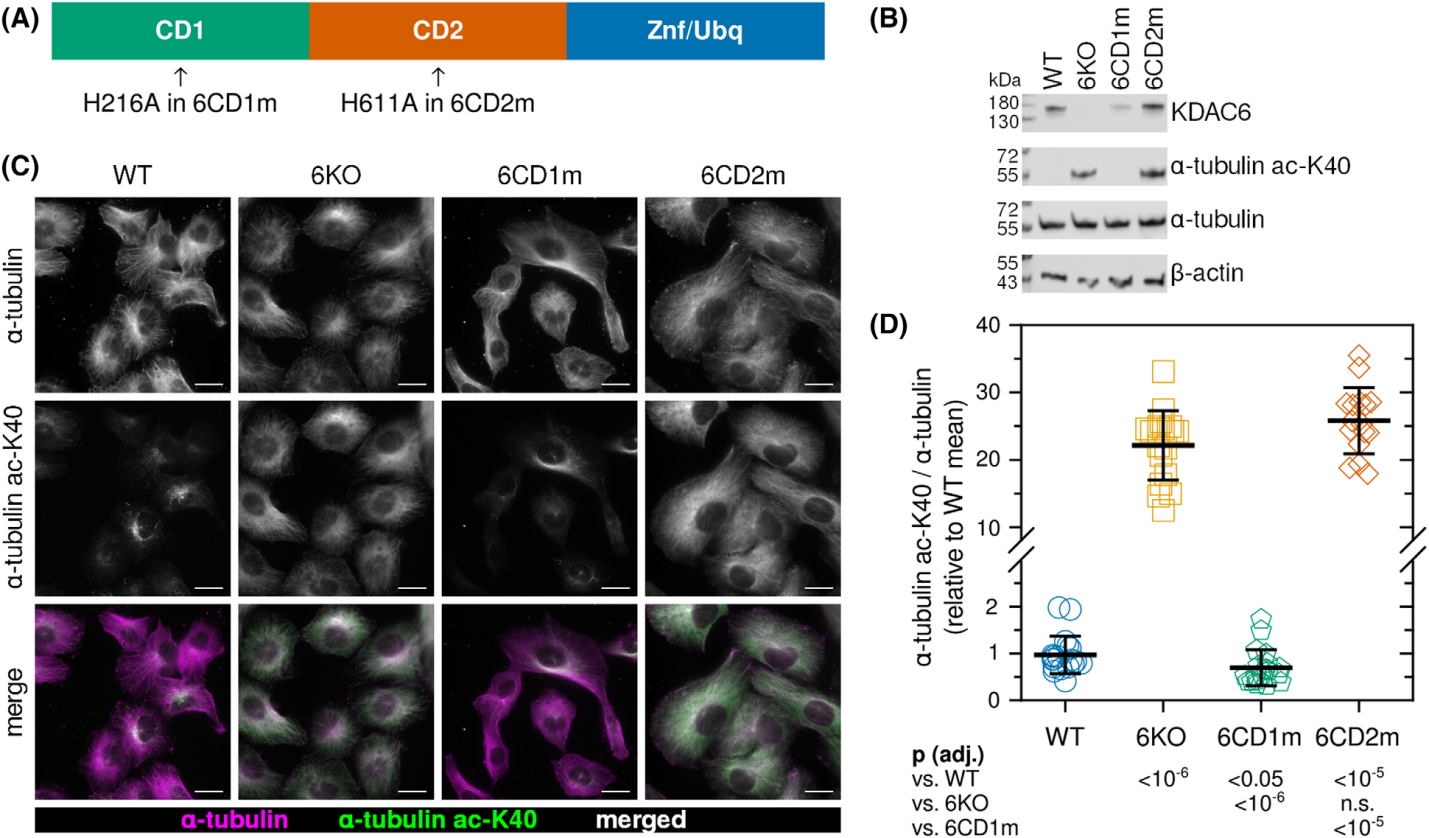
KDAC6 KO ac‐tubulin phenotype mimics KDAC6 CD2 inactivation. (A) Schematic of KDAC6 domains and inactivating mutations. (B) Representative immunoblot of lysates from each cell line comparing α‐tubulin ac‐K40 protein levels, as well as KDAC6 presence or absence. β‐actin serves as a loading control. (C) Representative immunofluorescence for each cell line showing total α‐tubulin (magenta in merge) and α‐tubulin ac‐K40 (green in merge) in fixed cells for each cell line. Scale bars represent 20 μm. Image intensities were individually maximized without cropping to emphasize distribution rather than relative abundance. (D) The ratio of α‐tubulin ac‐K40/α‐tubulin immunofluorescence intensities for individual cells, normalized to the wild‐type mean (*n* ≥ 16 for each group). Bars represent mean and standard deviation, and *P* values were determined by Mann–Whitney *U* tests.

Based on the known role of KDAC6 CD2 in α‐tubulin deacetylation and our previous gene expression data implicating CD2 in cell motility and cytoskeletal organization, we hypothesized that inactivating KDAC6 CD2 would lead to changes in cell motility [51]. To compare the existing 6CD1m and 6CD2m cells (Fig. 1A) to total loss of KDAC6 function, we used an analogous CRISPR/Cas9‐based method to create a KDAC6 knockout (6KO) in the same HT1080 parent cell line. To ensure that KDAC6 protein was not present in the 6KO line, we performed an immunoblot, which demonstrated there was no detectable KDAC6 in the 6KO line, even after overexposure (Figs 1B and S1). As expected, loss of KDAC6 in the 6KO line led to hyperacetylation of α‐tubulin at K40 (Fig. 1B,C). 6KO cells demonstrated a > 20‐fold increase in the immunofluorescence ratio of α‐tubulin acetylated at K40 (α‐tubulin ac‐K40) to total α‐tubulin compared to WT cells (Fig. 1D). This dramatic increase in α‐tubulin ac‐K40 is not significantly different from the increase observed in 6CD2m cells. Although the expression of the mutated KDAC6 in the 6CD1m cells is qualitatively lower than that of the WT and 6CD2m lines (Fig. 1B), this result does not lead to an increase in α‐tubulin acetylation (Fig. 1B,C) and is consistent with our initial characterization of the 6CD1m and 6CD2m lines [51].

To evaluate which cellular processes were perturbed by elimination of KDAC6, we performed RNA‐seq analysis of the 6KO cell line to identify gene expression changes compared to WT HT1080 cells (Table S2). Notably, GO enrichment analysis of downregulated genes identified statistically significant terms related to cell motility in 6CD2m, but not in the 6KO line (Tables 1 and S2). However, the 6KO line had upregulated genes associated with many actin processes, in contrast to the few such processes associated with downregulated genes in 6CD2m and no correlation in 6CD1m. Overall, the differences in GO terms suggest that at least KDAC6 CD2 has a role in cell motility and that KDAC6 activity may influence actin structure.

**Table 1:** Direction of gene expression changes associated with selected GO terms for each cell line.

GO term	GO ID	6KO	6CD1m*	6CD2m*
Cell motility	GO:0048870			Down
Regulation of locomotion	GO:0040012			Down
Negative regulation of locomotion	GO:0040013			Down
Regulation of cell motility	GO:0030334	Up		Down
Negative regulation of cell motility	GO:0030336			Down
Positive regulation of cell motility	GO:0030335			Down
Regulation of cell shape	GO:0008360			Down
Actin filament‐based process	GO:0030029	Up		Down
Actin filament organization	GO:0007015	Up		
Regulation of actin filament organization	GO:0110053	Up		
Actin cytoskeleton organization	GO:0030036	Up		
Actin filament depolymerization	GO:0030042	Up		
Regulation of actin filament‐based process	GO:0032970	Up		Down
Regulation of actin cytoskeleton organization	GO:0032956	Up		Down
Regulation of actin filament bundle assembly	GO:0032231	Up		
Regulation of actin filament length	GO:0030832	Up		
Regulation of actin polymerization or depolymerization	GO:0008064	Up		
Regulation of actin filament depolymerization	GO:0030834	Up		
Positive regulation of actin filament depolymerization	GO:0030836	Up		

* [51].

### KDAC6 CD1 and CD2 have opposing effects on cell motility

**Fig. 2:**
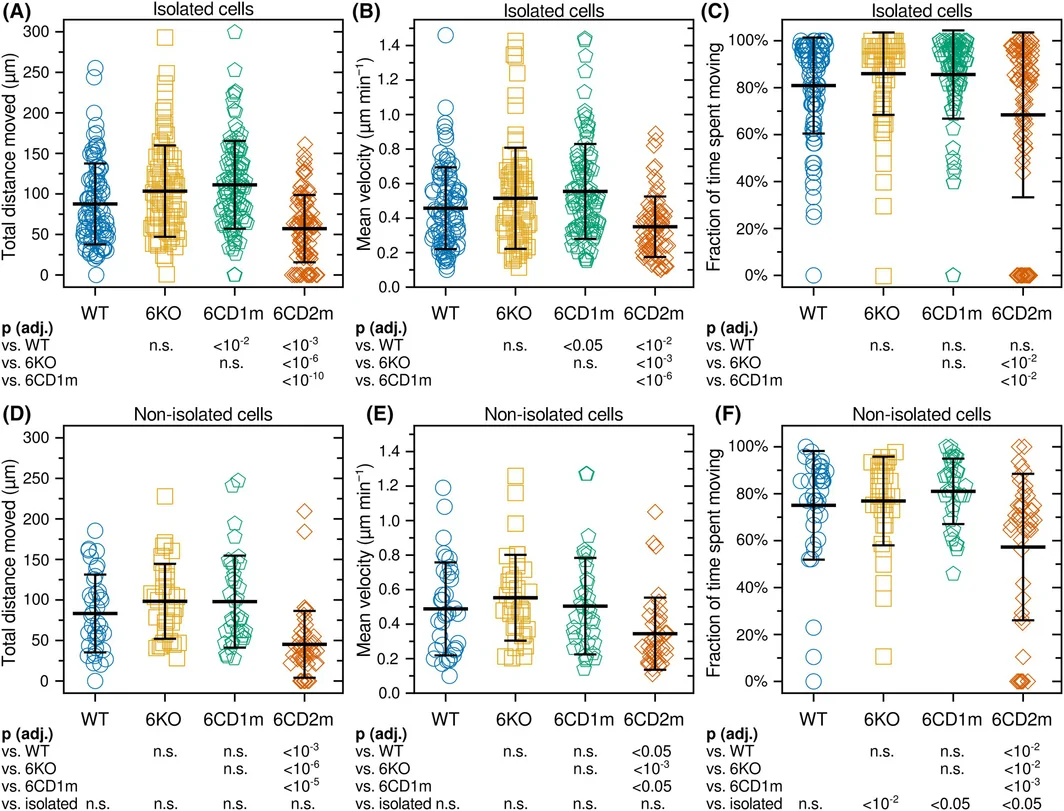
KDAC6 CD1 and CD2 inactivation cause opposite changes to cell motility. Individual cells were tracked for each cell line for 4 h and tracks were analyzed to quantitatively assess cell movement. (A) Total distance traveled by each isolated cell for each cell line (*n* ≥ 88 for each cell line). (B) For the time spent moving, mean velocity for each isolated cell (*n* ≥ 72 for each cell line). (C) For each isolated cell, the fraction of time the cell was actively moving (*n* ≥ 88 for each cell line). (D) Total distance traveled by each non‐isolated cell for each cell line (*n* ≥ 36 for each cell line). (E) For the time spent moving, mean velocity for each non‐isolated cell (*n* ≥ 36 for each cell line). (F) For each non‐isolated cell, the fraction of time the cell was actively moving (*n* ≥ 36 for each cell line). Bars represent mean and standard deviation, and *P* values were determined by Mann–Whitney *U* tests.

To assess the effect of KDAC6 activity on cell motility, we used time‐lapse imaging of the migration of single cells within a sparsely populated area for each of the cell lines. Cell movement was tracked for individual cells over 4 h (Fig. S2), and tracks were used to characterize cell motility. Initially, we analyzed only isolated cells, defined as cells that were not touching any other cells during the tracking window. First, we measured the total distance individual cells moved during the experiment (Fig. 2A). This was quite variable across different cells in the population, even within the same cell line, as the distance traveled by WT cells ranged from 0 μm to greater than 250 μm. The distance covered by 6KO cells was not statistically different from WT cells. In contrast, the mean distance moved by 6CD1m cells was 26% greater than that of WT cells and statistically significant. Conversely, 6CD2m cells traveled significantly less than the other cell lines, and only half the average distance of 6CD1m cells. Notably, there were more 6CD2m cells that did not move at all, and the maximum distance traveled by any 6CD2m cell was only about 60% of the maximum distance of the other cell lines during the observation window (Fig. 2A). Next, we calculated the mean velocity of individual cells, considering only the portion of time when they were actively moving (Fig. 2B). The trends were the same as for the total distance traveled. 6KO cells were similar to WT, 6CD1m cells moved significantly faster than WT, and 6CD2m cells moved significantly slower than all of the other cell lines (Fig. 2B). Finally, as many of the cells did not move constantly during the observation window, we determined the fraction of time each cell spent actively moving (Fig. 2C). Here, we found that although the mean was significantly lower for the 6CD2m cells, the difference in time spent moving between WT and 6CD2m cells was not significant; however, the 6CD2m cells spent significantly less time moving than did 6KO and 6CD1m cells (Fig. 2C).

To evaluate whether the motility patterns were specific to isolated cells, we also investigated the movement of nonisolated cells, using cells that were adjacent to at least one other cell at the beginning of the 4‐h observation period. We analyzed several nonisolated cells for the same parameters considered for isolated cells (Fig. 2D–F). For nonisolated cells, the overall trends were the same; however, there was no significant difference between WT, 6KO, and 6CD1m cells with respect to any of the parameters measured. As seen with isolated cells, 6CD2m nonisolated cells were significantly less motile than each of the other cell lines, measured by less total distance traveled, lower mean velocity, and a smaller fraction of time spent moving (Fig. 2D–F). Finally, we considered whether cells in each cell line behaved differently when they were adjacent to other cells, compared to when they were isolated. We hypothesized that cells would be less motile when they were touching one another. For WT cells, we found that there were no significant differences in the parameters we measured when comparing isolated to nonisolated cells. For each of the mutant cell lines, we observed a significant difference in the fraction of time spent moving: 6KO, 6CD1m, and 6CD2m cells spent 5–10% less time moving when they were not isolated (Fig. 2C,F). There were no significant differences in distance traveled or mean velocity when comparing isolated and nonisolated cells of each cell line (Fig. 2).

Overall, inactivation of KDAC6 CD2 resulted in cells that moved less and more slowly than WT cells, while inactivation of KDAC6 CD1 led to cells that moved slightly but significantly faster than WT cells. Intriguingly, eliminating KDAC6 negated these effects, as the 6KO cell line was not significantly different from either the WT line or the 6CD1m line for any of the parameters measured (Fig. 2). To ensure that these results were not specific to unguided motility in low‐density cells, we also used a scratch assay to characterize bulk migration rates of these cell lines. We observed the same trend in migration speed for all mutant cell lines with respect to WT cells (Fig. S3). The migration phenotype did not correlate with α‐tubulin ac‐K40 levels, as both 6KO and 6CD2m cells had obvious increases in acetylated α‐tubulin levels compared to WT (Fig. 1B,C), but only 6CD2m cells had impaired migration speed. This outcome is consistent with the GO analysis, in which only 6CD2m downregulated genes were associated with cell motility (Table 1). Thus, we conclude that KDAC6 CD1 and CD2 each play distinct and opposing roles in cell motility in HT1080 cells, and the function is not solely mediated through α‐tubulin acetylation.

### Inactivation of KDAC6 CD2, but not knockout, leads to distinct cell morphology

**Fig. 3:**
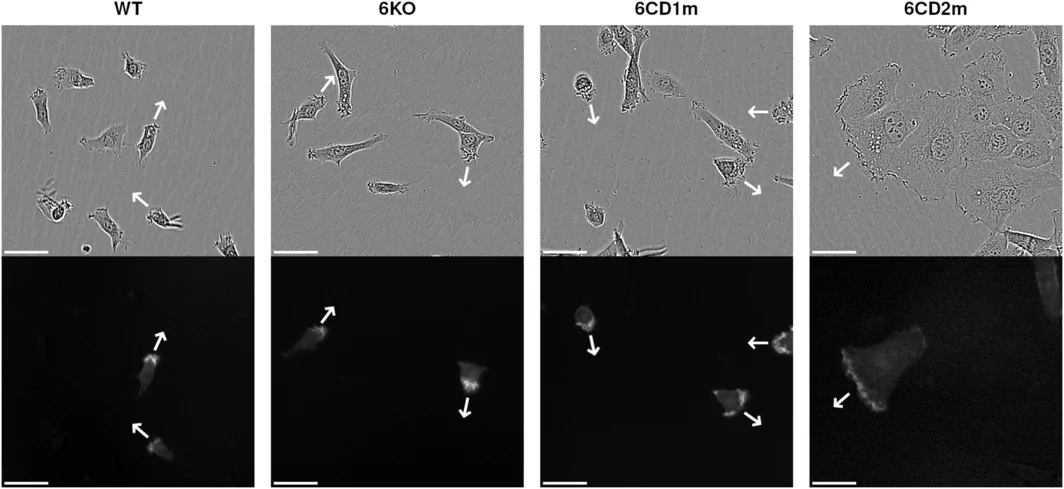
Inactivation of KDAC6 domains results in distinctive morphology and F‐actin distribution at the leading edge. Representative phase contrast (top) and Lifeact‐GFP (bottom) images for each cell line. Arrows indicate direction of motion, as further illustrated in Movies S1– S4. Scale bars represent 50 μm. All phase contrast image intensities were normalized to a common range with cropping of less than 1% of the total signal. Fluorescence image intensities were individually maximized with a fixed background subtraction to emphasize distribution rather than relative abundance.

**Fig. 4:**
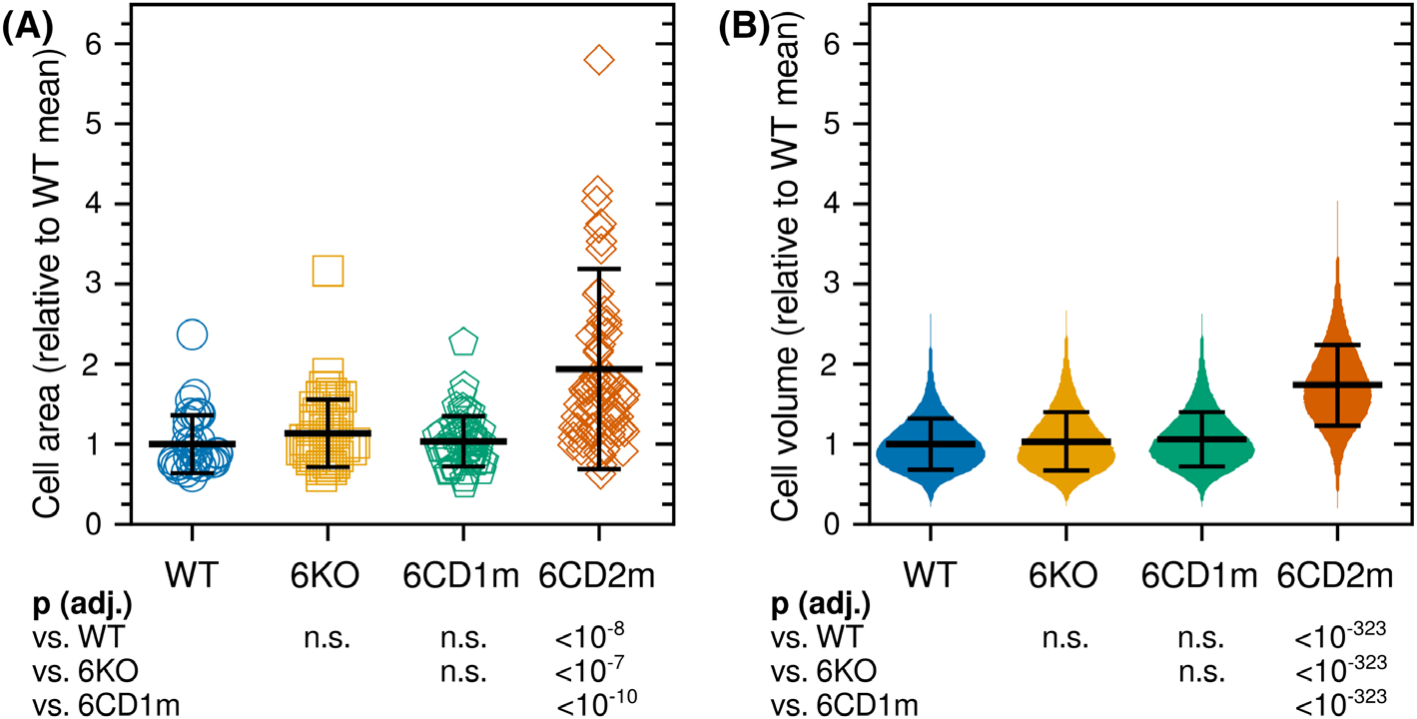
6CD1m and 6CD2m cells have distinct morphology. (A) The relative areas of individual cells (*n* ≥ 36 cells per line). Images of immunofluorescence of F‐actin in fixed cells from each cell line were used to measure the area of individual cells grown on plasma‐treated glass coverslips. Cell areas were normalized such that the mean area of wild‐type cells (861 μm^2^) was defined as 1. (B) Relative cell volume as determined using flow cytometry front scatter area (*n* > 930 000 cells per line). The enclosed areas represent individual values binned in increments of 500. Mean wild‐type volume was defined as 1. Bars represent mean and standard deviation, and *P* values were determined by Mann–Whitney *U* tests.

In addition to being less motile, 6CD2m cells had a different morphology than WT HT1080 cells. In both live and fixed cells, the 6CD2m cells appeared larger and more spread out than the other cell lines (Figs 1C and 3, Movies S1– S4). We also frequently observed 6CD2m cells in monolayer patches, whereas the cells of other cell lines interacted only transiently with each other. It was also apparent that 6CD2m cells tended to retain attachment to neighboring cells even when motile (Movie S4). To measure the difference in cell spreading, we used the immunofluorescence images with which we visualized F‐actin in the cell lines to measure the cell area of individual cells from each cell line (Fig. 4A). 6KO and 6CD1m cells were similar in area to WT. Conversely, 6CD2m cells were, on average, double the area of WT and 6CD1m cells (Fig. 4A). Thus, inactivation of CD2 had a significant effect on cell area, while there was no significant effect upon CD1 inactivation or KDAC6 knockout. Flow cytometry revealed that the 6CD2m cells were also significantly and substantially larger than WT cells by volume, while all the other cell lines were similar to each other (Fig. 4B). The 75% increase in cell volume of 6CD2m cells does not appear to be due to generalized cell cycle defects, as 6CD2m cells have a DNA content distribution that is not statistically different from WT (Fig. S4). Assuming a constant cell height for WT and 6CD2m cells, the cell volume results suggest that the average area of 6CD2m cells on a surface should be approximately 75% larger than that of WT cells. As we observe that 6CD2m cells are actually 100% larger than WT cells on average, we extrapolate that 6CD2m cells have an average height approximately 15% less than that of WT cells. We acknowledge that HT1080 cells are not uniformly distributed in area and that follow‐up studies by confocal microscopy would be necessary to thoroughly characterize the variation in cell height on a surface, but the relationship between the volume and area measurements is consistent with the flatter appearance of 6CD2m cells in phase contrast images (Fig. 3). Together, these results indicated that loss of KDAC6 CD2 activity led to larger cells that spread flatter over the surface than the other cell lines and appear to maintain more cell–cell contacts.

### Inactivation of KDAC6 catalytic domains leads to distinct F‐actin distributions

**Fig. 5:**
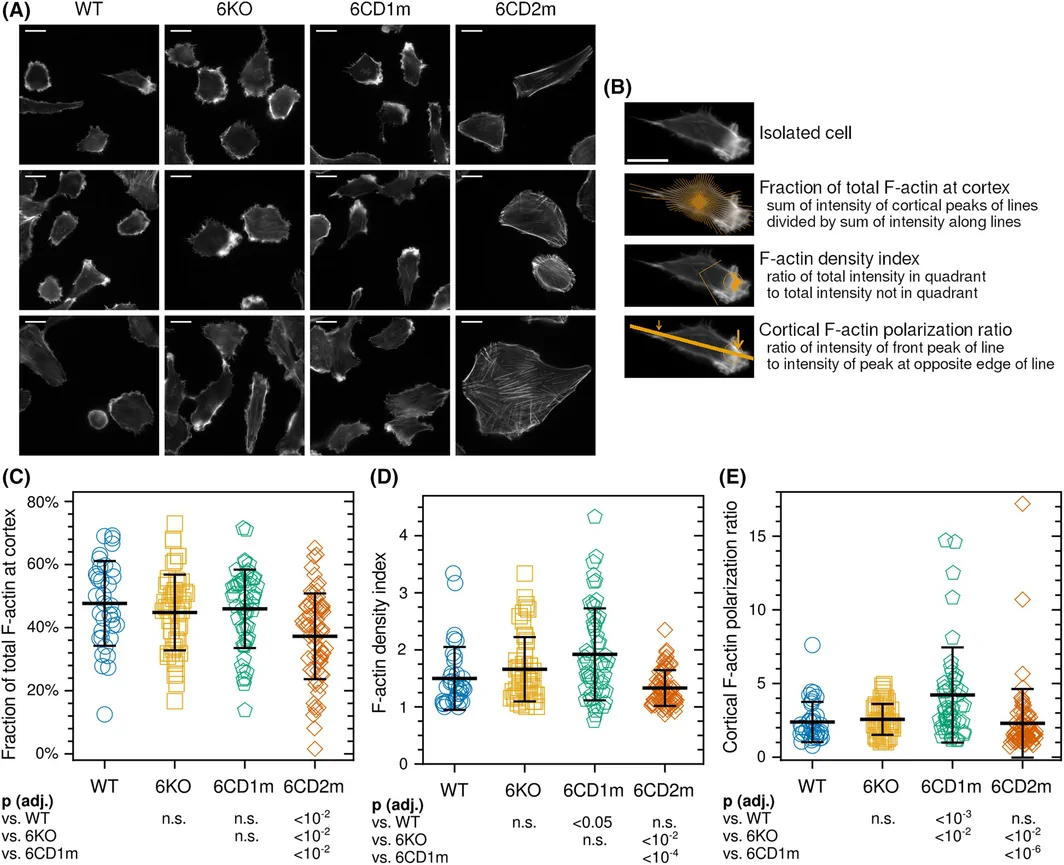
Inactivation of KDAC6 CD1 and CD2 lead to distinct F‐actin distributions. (A) Representative images of HT1080 wild‐type and derivative cell lines grown on glass coverslips, fixed, labeled with fluorophore‐conjugated phalloidin to stain F‐actin, and subjected to fluorescence microscopy. Scale bars represent 20 μm. Image intensities were individually maximized without cropping to emphasize distribution rather than relative abundance. (B) Schematic for the analysis of F‐actin distributions in cells. Individual isolated cells such as those displayed in A were subjected to processing, allowing quantitative analysis and comparisons of F‐actin distribution between the cell lines. (C) The fraction of total F‐actin at the cortex for individual cells in each cell line. Cortical F‐actin analysis was performed as indicated in B. (D) The F‐actin density index, determined as described in B to measure the fraction of F‐actin concentrated in one quadrant of the cell, using the same cells as in C. A higher number indicates more concentrated F‐actin. (E) The cortical F‐actin polarization ratio, determined as described in B to measure the ratio of F‐actin concentrated at the cortex versus the opposing side of the cell, using the same cells as in C. A higher number indicates more concentrated cortical F‐actin. For each analysis and cell line, *n* ≥ 36. Bars represent mean and standard deviation, and *P* values were determined by Mann–Whitney *U* tests.

The lack of correlation of the α‐tubulin ac‐K40 status with either the motility or morphology phenotypes among the 6KO and 6CD2m cell lines led us to investigate whether these phenotypes may be driven by the F‐actin distribution in these cells, as suggested by the numerous actin GO terms from the gene expression analysis (Table 1). Using fixed phalloidin‐stained cells, we observed that there appeared to be less cortical F‐actin in the 6CD2m cell line (Fig. 5A). To quantitatively analyze the differences in F‐actin distribution between the cell lines, we used automated analyses to analyze the F‐actin distribution for many individual cells from each cell line (Fig. 5B), focusing exclusively on isolated cells to avoid artifacts resulting from cell–cell contacts. Indeed, the 6CD2m cells had significantly less of their F‐actin in the cortical region of the cell than did the other cell lines. The fraction of F‐actin cortically localized in 6KO and 6CD1m cells was the same as WT (Fig. 5C). We also considered the average density of F‐actin, resulting in the F‐actin density index, to evaluate whether the distribution of F‐actin was more highly concentrated in the quadrant of the cell having the most intense region of cortical F‐actin, independent of the total amount of cortical F‐actin (Fig. 5B). Due to the irregular shape of HT1080 cells, we found that using the mean signal of a quadrant from the center of the nucleus toward the most intense region of cortical F‐actin provided the most robust metric of altered cellular distribution and most reliably resulted in a zone that incorporated the entire portion of the cortex with enhanced F‐actin. Results of the F‐actin density index analysis, for which a higher value indicates a greater average concentration of F‐actin in the quadrant of the cell containing the most intense region of cortical F‐actin, revealed that 6CD1m cells had more concentrated regions of F‐actin compared to WT cells (Fig. 5D). There was no difference in the F‐actin density index for either 6KO or 6CD2m compared to WT cells; however, the density in 6CD2m cells was significantly lower than either 6CD1m or 6KO cells. To evaluate the overall polarization of the F‐actin in each cell line specifically at the cortex, we calculated the polarization ratio of the intensity of the most concentrated cortical F‐actin to the intensity of cortical F‐actin at the opposite edge of the cell along a straight line (Fig. 5B). 6CD1m is significantly more polarized than all other lines, with a ratio approximately double that of WT (Fig. 5E). This result indicates that 6CD1m cells are more likely to have cortical F‐actin that is highly concentrated in a small portion of the cell, and is consistent with the density index analysis. While F‐actin polarization in 6CD2m cells was not different than in WT cells, F‐actin in 6CD2m cells was significantly less polarized than in 6CD1m or 6KO cells (Fig. 5E). Overall, inactivation of 6CD1 and 6CD2 led to opposite effects with regard to F‐actin distribution, consistent with our observations of cell motility. Furthermore, the fraction and concentration of cortical F‐actin positively correlate with motility across the cell lines analyzed here.

Because our quantitative analysis was conducted in fixed cells, we independently confirmed that the cortical F‐actin regions that we visualized in fixed cells corresponded to the leading edge of migrating cells in each cell line. These experiments utilized GFP‐Lifeact, which binds to F‐actin, allowing visualization of actin structures in live cells [59]. Qualitatively, F‐actin was concentrated at the leading edges in time‐lapse imaging of migrating cells (Fig. 3, Movies S1– S4). We did not observe morphological or migration differences between cells transfected with GFP‐Lifeact and untransfected cells in the same well for any of the cell lines (Movies S1– S4), indicating that our observations are likely not impacted by reported effects of high dosing of the Lifeact peptide [61, 62]. Overall, our data suggest that the extent of cortical F‐actin aggregation appeared to correlate with the observed motility behaviors (Fig. 2). Thus, it is plausible that inactivation of KDAC6 CD1 or CD2 led to increased or decreased migration, respectively, in response to changes in the cellular actin distribution.

**Fig. 6:**
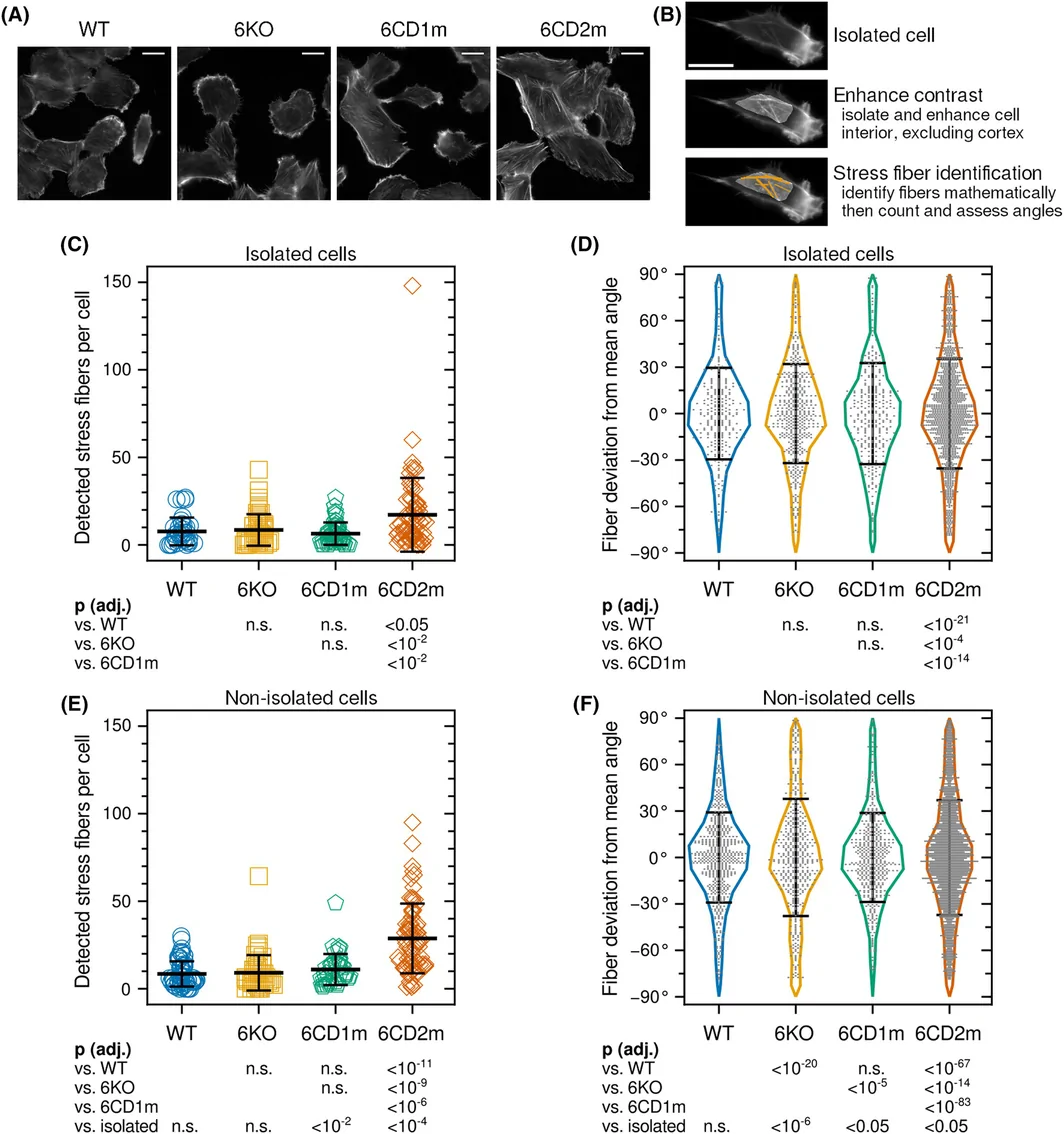
KDAC6 CD2 inactivation leads to an increased number of stress fibers and reduced fiber alignment. (A) Representative image of fixed nonisolated cells stained with fluorophore‐conjugated phalloidin for each cell line. Scale bars represent 20 μm. Image intensities were individually maximized without cropping to emphasize distribution rather than relative abundance. (B) Schematic for the analysis of stress fibers. Analysis was performed separately on isolated and non‐isolated cells to identify and analyze stress fibers. (C) The count of stress fibers detected in each isolated cell in each cell line (*n* ≥ 36 cells for each cell line). (D) Stress fiber alignment assessed by the deviation of each individual stress fiber from the mean fiber angle of the cell containing the fiber (*n* ≥ 259 fibers for each cell line). For each isolated cell with at least 4 stress fibers detected, a mean angle for the cell was calculated and set to 0°. Dots represent the angle deviation from the mean for each individual stress fiber. The outlines represent angle deviations binned in increments of 15° as used for statistical testing. (E) Stress fiber count as described in C for individual non‐isolated cells (*n* ≥ 43 cells for each cell line). (F) Stress fiber alignment in non‐isolated cells (*n* ≥ 453 fibers for each cell line), as described in D. Bars represent mean (except angle plots) and standard deviation, and *P* values were determined by Mann–Whitney *U* tests (counts) or chi‐squared tests (angles).

In addition to the larger area of the 6CD2m cells, they also contained striking stress fibers compared to the other cell lines, both as isolated cells (Fig. 5A) and even more so when in contact with other cells (Fig. 6A). Using the same set of individual, isolated cells that we used for the cortical F‐actin analysis, we identified and analyzed the stress fibers in each of the cell lines (Fig. 6B). 6CD2m cells averaged more than twice as many stress fibers per cell than any of the other cell lines (Fig. 6C). In addition to the increased number of stress fibers, the stress fibers in 6CD2m cells appeared less aligned compared to the other cell lines (Figs 5A and 6A). To determine whether there was a quantitative change in stress fiber alignment in any of our cell lines, we set the average angle of the fibers within each cell to 0° and plotted the deviation in angle of each fiber from the average. We found that upon inactivation of CD2, the distribution of angles was less centered around the mean, indicating that the stress fibers in the 6CD2m line were significantly less aligned than in the other cell lines (Fig. 6D). We also performed the same analysis on stress fibers in nonisolated cells from each of the cell lines, to investigate whether cell–cell contacts meaningfully impacted stress fiber quantity or alignment. As with isolated cells, the nonisolated 6CD2m cells had an increased quantity and reduced alignment of stress fibers compared to the other lines (Fig. 6E,F). The differences between 6CD2m and the other cell lines were even more pronounced in non‐isolated cells than in the isolated cells, despite the minimal change in motility patterns for isolated and nonisolated cells (Fig. 2). This analysis confirmed that inactivation of KDAC6 CD2 resulted in an increase in stress fibers and a decrease in alignment among those fibers.

Overall, these data demonstrate that 6CD1m cells had more concentrated cortical F‐actin. Conversely, 6CD2m cells tended to be more spread and much of their F‐actin was in poorly aligned stress fibers throughout the cell, rather than accumulated at the cortex. The trend of increasing concentration of cortical F‐actin from 6CD2m to WT to 6CD1m positively correlated with the observed migration rates. 6KO cells were most similar to WT cells with regard to actin distribution; however, they were also not significantly different from 6CD1m cells (Figs 5 and 6), consistent with the migration and morphology data (Figs 2 and 4).

### Changes in localization of cortactin and heat shock protein 90 do not correlate with KDAC6‐dependent phenotypes

**Fig. 7:**
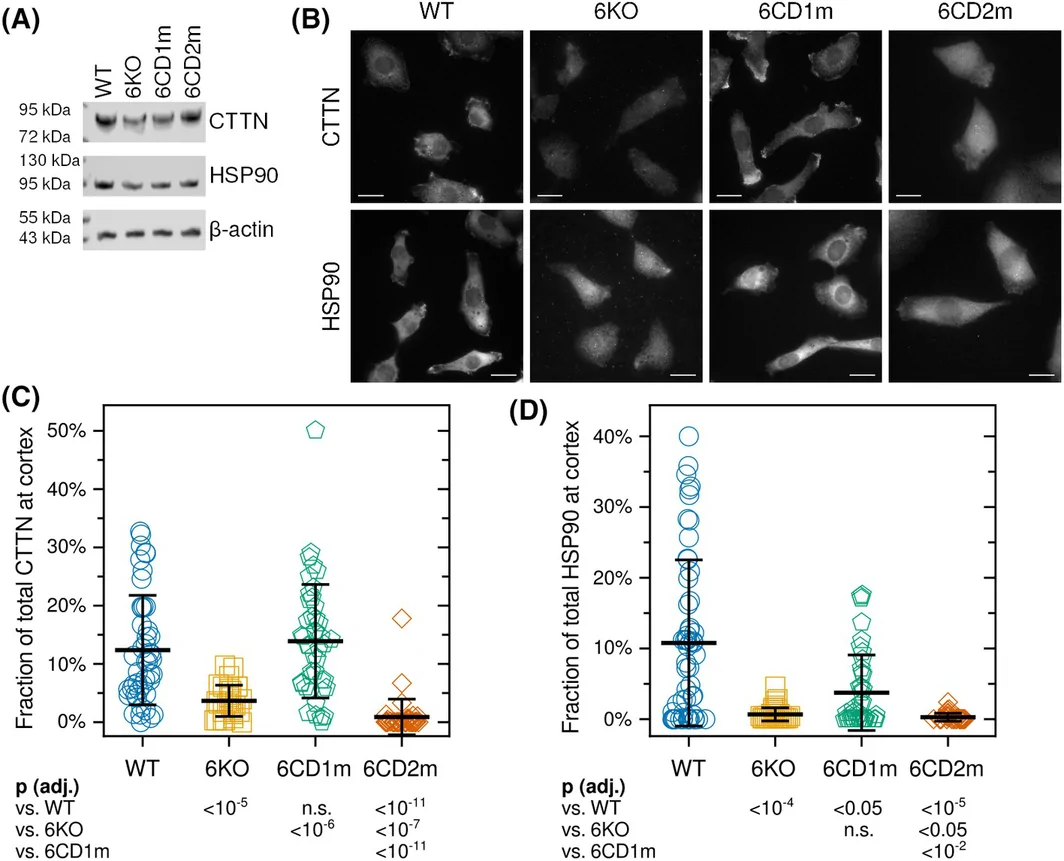
KDAC6 mutant cell lines have reduced cortical localization of actin‐associated proteins. (A) Representative immunoblot of lysates from HT1080 and HT1080‐derived cell lines comparing expression levels of CTTN and HSP90. β‐actin serves as a loading control. (B) Representative immunofluorescence images for each cell line of cells grown on glass coverslips, fixed, and probed for CTTN or HSP90. Scale bars represent 20 μm. Image intensities were individually maximized without cropping to emphasize distribution rather than relative abundance. The fraction of total (C) CTTN and (D) HSP90 in individual, isolated cells at the cortex for each cell (*n* ≥ 30 cells for each cell line and protein). Bars represent mean and standard deviation, and *P* values were determined by Mann–Whitney *U* tests.

To investigate whether the changes in F‐actin distribution were mediated through proteins previously associated with both KDAC6 and cell motility, we evaluated localization of CTTN and HSP90, which both co‐localize with cortical actin and can be acetylated [3, 34]. Total protein levels of CTTN and HSP90 were qualitatively similar in all cell lines, indicating that loss of KDAC6 catalytic function did not greatly affect the levels of these proteins in cells (Figs 7A and S5). To look at localization of CTTN and HSP90, we performed immunofluorescence in each cell line (Fig. 7B). For each protein, we measured the percent of the total protein found in the cortical region of cells in each cell line using the same method as for measuring cortical F‐actin (Fig. 5B). Compared to WT cells, there was no change in the 6CD1m cell line with respect to CTTN distribution, but we observed significantly reduced cortical CTTN in both the 6KO and 6CD2m cell lines (Fig. 7C). Interestingly, the decreased CTTN localization in the 6KO and 6CD2m cell lines does not correlate with the motility, morphology, and F‐actin distribution defects, as those were not observed in 6KO cells (Figs 2, 3, 4, 5). We found that the proportion of total HSP90 signal found at the cortex was significantly reduced in all three mutant cell lines compared to WT cells. This was most dramatic in 6CD2m and 6KO cells, in which there was a notable absence of HSP90 at the cortex (Fig. 7D). For all three mutant lines, the median HSP90 signal at the cortex was less than 1%, and an order of magnitude below WT (Table S1). Thus, HSP90 localization did not correlate with either cortical F‐actin or motility phenotypes, as all perturbations of KDAC6 resulted in a significant reduction of cortical HSP90. Together, loss of KDAC6 activity did affect cortical localization of CTTN and HSP90, two putative KDAC6 substrates involved in cell migration; however, these effects did not correlate with the observed migration phenotypes, suggesting that they are not the drivers of the KDAC6‐dependent motility phenotypes that we have observed.

### Inactivation of KDAC6 CD2 correlates with enhanced focal adhesions

**Fig. 8:**
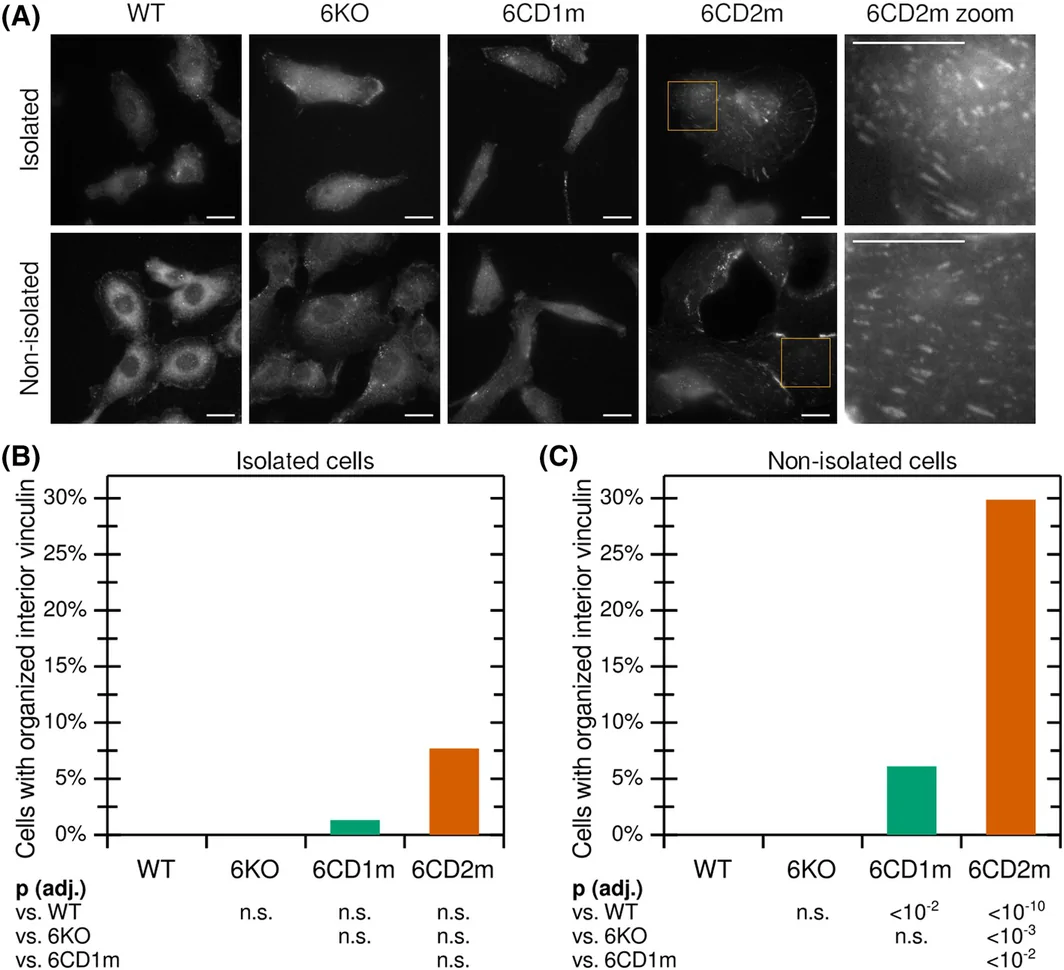
KDAC6 CD2 inactivation is associated with an enhanced presentation of focal adhesions. (A) Representative immunofluorescence images for each cell line of cells grown on glass coverslips, fixed, and probed for VCL. Scale bars represent 20 μm. Image intensities were individually maximized without cropping to emphasize distribution rather than relative abundance. Zoom panels are examples of organized interior VCL. (B) Proportion of isolated cells having organized interior VCL, excluding consideration of the immediate cortical region (*n* ≥ 42 cells for each cell line). (C) Proportion of non‐isolated cells having organized interior VCL, excluding consideration of the immediate cortical region (*n* ≥ 51 cells for each cell line). Fisher Exact tests were used to determine *P* values.

The striking change in cell morphology and increase in stress fibers in the 6CD2m cell line led us to investigate whether these cells perhaps also contained increased focal adhesions. Notably, we previously reported gene expression changes of genes involved in cell adhesion in the 6CD2m cell line [51]. To investigate, we probed the distribution of VCL in each cell line, as a marker of focal adhesions. Some cells in all lines presented with focal adhesions near the cortex (Fig. 8A), but these were frequently distributed around the cell rather than localized to a small region of the cortex, and likely associated with cell spreading rather than cell motility. Therefore, we were not able to draw any conclusions about variation in cortical focal adhesions from the fixed images. However, it was apparent that the 6CD2m cell line had more cells with focal adhesions distributed throughout the cell than did the other cell lines, which was significant for non‐isolated cells (Fig. 8). This result is consistent with the greater density of F‐actin stress fibers in the same population (Fig. 6). As with the other phenotypes we have observed here, eliminating KDAC6 did not result in an increase in focal adhesions throughout the cells, reinforcing that hyperacetylation of α‐tubulin is not driving the change in focal adhesions. Instead, multiple domains of KDAC6 are contributing to the regulation of cell morphology and motility.

## Discussion

Inactivation of KDAC6 CD1 resulted in an increased average concentration of cortical F‐actin at the leading edge. Functionally, 6CD1m cells had an increased migration velocity relative to WT HT1080 cells, indicating that KDAC6 CD1 is a negative regulator of motility. As our experimental system isolated the role of KDAC6 CD1, we were able to uncover this previously unknown function that was obscured in prior work using knockouts, knockdowns, and inhibitors not specifically targeted to CD1. This finding is significant, as there has only been one reported cellular target of KDAC6 CD1, and that target has not been annotated as being associated with our observed phenotypes in either Uniprot or GO [63]. Furthermore, structural data have revealed that CD1 and CD2 have distinctly shaped active sites, which presumably drives the narrower and distinct substrate preferences of CD1 *in vitro* [23]. The phenotype described here for the 6CD1m line strongly suggests that there is an unidentified target of KDAC6 CD1 that modulates actin distribution to affect cell motility.

In contrast, inactivation of KDAC6 CD2 resulted in larger, flatter cells, less cortical F‐actin and an increased number of stress fibers, which were less aligned compared to WT HT1080 cells. Functionally, 6CD2m cells migrated slower and moved less frequently. The impaired motility in the 6CD2m cell line is consistent with our prior observation that genes associated with cell motility were downregulated in this cell line [51]. The shift in F‐actin organization in the 6CD2m cell line from cortical patches to increased stress fibers is likely driving the less motile phenotype, although we have not resolved whether formation of stress fibers is a cause of the reduced cortical F‐actin. Intriguingly, the gene expression changes associated with GO adhesion terms in the 6CD2m cell line, as well as the increase in focal adhesions throughout these cells compared to the other cell lines, suggest that the stress fiber organization may reflect an underlying change in regulation of focal adhesions [51]. Overall, our observations are consistent with a model in which KDAC6 CD2 activity alters the cellular actin distribution and ultimately promotes cell motility.

Overall, our results have demonstrated that the catalytic domains of KDAC6 each play a distinct role in regulating cell motility and morphology through the actin cytoskeleton. Inactivation of KDAC6 CD1 and CD2 led to opposite effects on cell motility: Inactivation of CD1 led to increased motility, while inactivation of CD2 led to decreased motility. CD1 and CD2 inactivation also led to opposite effects on F‐actin distribution, and the arrangements of F‐actin in the various cell lines support the observed changes in motility. In fact, 6CD1m and 6CD2m cells were significantly different from one another in most metrics reported here, although only CD2 inactivation had a significant effect on morphology. Overall, we propose a model in which KDAC6 CD1 activity leads to reduced migration and reduced localization of actin to the cortex, whereas KDAC6 CD2 activity enhances migration, reduces cell spreading, increases localization of actin to the cortex, and promotes stress fiber alignment. Strikingly, the 6KO line was similar to WT HT1080 cells in both actin organization and motility, despite the dramatic increase in α‐tubulin ac‐K40. Although α‐tubulin ac‐K40 amounts are similar in 6CD2m and 6KO cells (Fig. 1), these cells have very different motility phenotypes (Figs 2 and 3 and Movies S2, S4). These data suggest that the role of KDAC6 in cell motility is at least partially independent of KDAC6 deacetylation of α‐tubulin K40. Furthermore, the target(s) of each domain are most likely distinct, as only CD2 inactivation, but not CD1 inactivation, resulted in a feedback mechanism leading to extensive changes in gene expression, as indicated by the lack of GO terms directly related to actin, motility, or morphology associated with CD1 inactivation (Table 1).

The lack of phenotype in 6KO cells suggests a potential mechanism arising from our data in which loss of CD1 and CD2, which alone cause opposite effects, result in little to no net change when combined. Unfortunately, we cannot directly test this idea while retaining endogenous KDAC6 expression, as attempts to make an analogous cell line in which endogenous KDAC6 is present but both CD1 and CD2 were inactivated did not produce a viable mutant cell line [51]. Comparisons to studies relying on overexpression, knockouts, or knockdowns are complicated because the third domain of KDAC6, which binds ubiquitin, has also been implicated as having a role in cell motility and actin structure in a non‐catalytic manner [30, 64]. Therefore, the loss of the third domain is likely contributing to the 6KO phenotype, and the apparent similarity to WT may not be due to a simple balancing of the effects of CD1 and CD2. However, a recent study examined the effects of inactivating the third domain in a manner analogous to our approach, in lymphocytes, and the gene expression changes resulted in no actin‐related GO terms and only two motility and locomotion terms (Table S3), suggesting that the third domain has a relatively minor role in the phenotypes assessed here [55]. The same study also observed that the KDAC6 knockout was more similar to WT than the third domain mutant cell line, consistent with the behavior of the 6KO cell line. Despite the caveats of comparing to prior work manipulating expression of the entire protein, and between lymphocyte motility to fibroblast‐like motility that may confound amoeboid and mesenchymal migration modes, several previous studies do provide relevant context. Overexpression of WT or catalytically inactive KDAC6 in mouse fibroblast cells demonstrated a requirement for the catalytic activity of KDAC6 in promoting cell motility, consistent with the function that we found for CD2 [20]. A similar experiment performed in lymphocytes, in which either WT or a double mutant version of KDAC6 was overexpressed, resulted in faster migration in both circumstances, consistent with 6KO most closely resembling WT [30]. KDAC6 knockout experiments in MEFs and rhabdomyosarcoma cells were found to result in less cell migration in transwell migration and/or scratch assays, although effects were generally less than we observed upon inactivation of CD2 [29, 65]. Published observations of KDAC6 knockout in MEFs and human fibroblasts resulted in similar increases in cell spreading to what we have observed in the 6CD2m cells [66]. Another experiment in transformed fibroblasts revealed that knocking down KDAC6 modestly increased cell area to that of normal fibroblasts, thus negating the decrease in cell area and altered cell morphology associated with transformed, invasive cells [31]. Other groups reported similar decreases in migration using either KDAC6 knockdown or chemical inhibitors in a variety of mouse and human cell types, several of which were derived from human cancers [41, 45, 67, 68, 69, 70]. However, experiments using inhibitors may not recapitulate the phenotype of inactivation of a single KDAC because KDAC inhibitors, even those which are reported to be specific to a particular KDAC6, demonstrate significant off‐target effects [48, 49, 50]. Perhaps of particular importance, KDAC6 knockout mice developed with only a few reported abnormalities that did not impact growth or fertility despite having hyperacetylated tubulin, consistent with the observed minimal impact from complete loss of KDAC6 in this work and the analogous study on inactivation of the third domain [55, 71]. As prior work has not considered the role of each catalytic domain in isolation without also perturbing expression levels, the results described here add to the overall understanding of the role of KDAC6. However, limitations do arise from particular experimental approaches and cell lines. Thus, the general applicability of our conclusions, especially the cellular consequences of KDAC6 inactivation, will require further investigation.

With clearly established effects on motility and F‐actin reorganization for both catalytic domains of KDAC6, we aimed to understand how KDAC6 modulated the distribution of F‐actin in cells. Although actin is not an established substrate of KDAC6, other actin‐associated proteins are known to be acetylated. Of these, CTTN and HSP90 have both been proposed to be substrates of KDAC6 [34, 40, 45]. CTTN binds cortical F‐actin in cells and, in conjunction with several other actin‐associated proteins, is proposed to play a migration‐promoting role in cytoskeletal regulation by facilitating the formation and persistence of migratory structures such as lamellipodia [42, 72, 73, 74]. Furthermore, it has been reported that acetylation of CTTN impairs its actin‐binding ability in HeLa and HEK293T cells, and thus would impair motility [41, 46]. If KDAC6 was responsible for deacetylating CTTN and if this event caused increased migration, then we would predict that inactivation of KDAC6 might result in loss of cortical CTTN, as it would no longer be bound to cortical F‐actin and cause impaired motility. We did observe a decrease in cortical CTTN in the 6CD2m and 6KO cell lines (Fig. 7C). This suggests that KDAC6 CD2 is regulating the interaction of F‐actin and CTTN. However, the 6KO cell line does not demonstrate impaired motility (Fig. 2) or any change in cortical F‐actin compared to WT (Fig. 5), indicating that the interaction of CTTN and cortical F‐actin does not seem to be driving the motility phenotype that we have observed in HT1080 cells. Similarly, HSP90 has been reported to localize to membrane ruffles, and inhibition of HSP90 resulted in less ruffle formation and decreased cell migration. Thus, it has been proposed to facilitate cell motility by remodeling the actin cytoskeleton to promote membrane ruffling in MEFs, with KDAC6 deacetylation of HSP90 being an important regulator of this function [29]. However, our data do not support this model in HT1080 cells, as all three mutant cell lines led to a decrease in cortical localization of HSP90 (Fig. 7D), despite having a range of outcomes for change of cortical F‐actin (Fig. 5D). As each of the three mutant cell lines demonstrate distinct motility phenotypes compared to WT HT1080 cells, we conclude that any potential regulation of HSP90 by KDAC6 is not driving actin remodeling or cell motility. Unfortunately, we were not able to address whether the mutant KDAC6 proteins themselves had cellular distributions different than WT KDAC6, as commercial antibodies did not allow visualization of endogenous KDAC6 in our fixed cells.

The observed increase in focal adhesions throughout 6CD2m cells is consistent with existing models that there is potential crosstalk between microtubules and F‐actin through focal adhesions, with the potential for α‐tubulin K40 acetylation to influence this crosstalk. However, our data contrast with prior work describing a reduction in focal adhesions in the cell interior upon α‐tubulin hyperacetylation [38]. The differences between our work and the previous report may be due to the different cell types used (fibroblast‐like versus astrocytes), the method by which hyperacetylation was achieved, as well as our use of only rigid, uncoated surfaces versus the soft and rigid coated surfaces used in the prior study. In general, our approach of probing the function of single domains may also contribute to differences between the phenotypes we observe compared to other experimental systems probing KDAC6 function, as prior KDAC6 experiments have demonstrated that CD2 was only fully active when CD1 was intact, suggesting that CD1 may be regulating or contributing to the catalytic activity of CD2 [75, 76]. Similarly, other work has suggested a role for the third domain of KDAC6 in controlling cytoskeleton cross‐talk [64]. While these limitations are important caveats when potentially extrapolating the specific phenotypes reported here to other systems, these experiments do provide clear evidence that the two catalytic domains of KDAC6 independently regulate F‐actin distribution and impact cell motility.

Based on the stark differences between the cell lines analyzed here, particularly 6CD2m and 6KO, we hypothesize that KDAC6 CD1 and CD2 each have one or more KDAC6 targets that are unidentified and/or not known to play a role in F‐actin regulation that lead to the observed changes in cell morphology and motility. Our observations also suggest that KDAC6 may play a role in cell adhesion, and further investigation into a potential role for KDAC6 in regulating focal adhesions is also warranted. Such future studies will elucidate the mechanism by which each domain of KDAC6 exerts distinct effects on cytoskeleton organization, cell morphology, and motility.

## Conflict of interest

The authors declare no conflict of interest.

## Author contributions

TBT and TJW conceived the project. TBT, TJW, and TMH designed and supervised the experiments. ABP, CAR‐R, KEB, RHH, TBT, TMH, and TVJ performed the experiments. TBT, TJW, and TVJ analyzed the data. TBT wrote the initial draft. All authors reviewed and revised the manuscript.

## Supporting information


**Fig. S1.** Full immunoblots from Fig. 1. Full panels for all cropped blots in Fig. 1. The smaller band in the KDAC6 blot was consistent in proportional intensity relative to the larger band in several replicate blots from multiple lysate samples.
**Fig. S2.** KDAC6 variants demonstrate distinct single‐cell migration patterns. Time‐lapse images were collected for low‐density cells of (A) wild‐type, (B) 6KO, (C) 6CD1m, and (D) 6CD2m. The MTrackJ plugin for imagej was used to track the movement of individual, isolated cells for 4 h. The lines correspond to 50 representative tracks for each cell line.
**Fig. S3.** KDAC6 CD1 and CD2 inactivation cause opposite changes to bulk cell migration. (A) Representative images of scratch assay for each cell line to measure bulk migration rate. The blue area represents the scratched area containing no cells at 0, 2, and 4 h. Images at 8 h illustrate progress toward closure at a late time point. Scale bars represent 100 μm. All image intensities were normalized to a common range with cropping of less than 1% of the total signal. (B) Mean bulk velocity for each cell line (*n* ≥ 16 for each cell line). Images from scratch assay were analyzed to calculate the velocity of bulk cell movement using the change in position of the cell front represented by the edges of the blue area for each cell line during the first 4 h post‐scratch. Error bars represent standard deviation, and *P* values were determined by Mann–Whitney *U* tests.
**Fig. S4.** DNA content is not significantly changed in 6CD2m cells. The percentage of the total cell population having 2*n*, 4*n*, or an intermediate amount of DNA content in wild‐type and 6CD2m cells as measured by pooled flow cytometry data (≥ 6 independent experiments for each cell line; *n* ≥ 930 000 total events for each cell line). DNA content is labeled for each bar section. Error bars represent standard error of the mean of the independent experiments. The proportion of cells in each group is not statistically different (pairwise Mann–Whitney *U* tests, *P* > 0.05).
**Fig. S5.** Full immunoblots from Fig. 7. Full panels for all cropped blots in Fig. 7.

**Table S1.** Statistical descriptors for all data series.
**Table S2.** Significant gene expression changes in 6KO relative to wild‐type.
**Table S3.** Significant GO terms based on 6KO gene expression changes of false discovery rate < 0.001.

**Movie S1.** Representative example of motility behavior and F‐actin distribution (Lifeact‐GFP) for WT HT1080 cells, prepared as described for Fig. 3.

**Movie S2.** Representative example of motility behavior and F‐actin distribution (Lifeact‐GFP) for 6KO cells, prepared as described for Fig. 3.

**Movie S3.** Representative example of motility behavior and F‐actin distribution (Lifeact‐GFP) for 6CD1m cells, prepared as described for Fig. 3.

**Movie S4.** Representative example of motility behavior and F‐actin distribution (Lifeact‐GFP) for 6CD2m cells, prepared as described for Fig. 3.

## Data Availability

The data that support the findings of this study that are not included in their entirety in this report are openly available in Gene Expression Omnibus at www.ncbi.nlm.nih.gov/geo/, reference numbers GSE228549, GSE302260, and GSE302329, and in Open Science Framework at 10.17605/OSF.IO/Y6DSA (fixed cell fluorescence microscopy), 10.17605/OSF.IO/D9HSK (flow cytometry), and 10.17605/OSF.IO/S48WE (live cell microscopy).
