# Supplementary material for: KDAC6 alters cell morphology and motility through positive and negative modulation of F‐actin distribution
Source: FEBS Open Bio. 2026 Sep 25:10.1002/2211-5463.70302. Online ahead of print. doi: 10.1002/2211-5463.70302 (PMC13615371; doi:10.1002/2211-5463.70302)
Supplement: Supplementary file 1 [file FEB4-9999-0-s004.pdf]

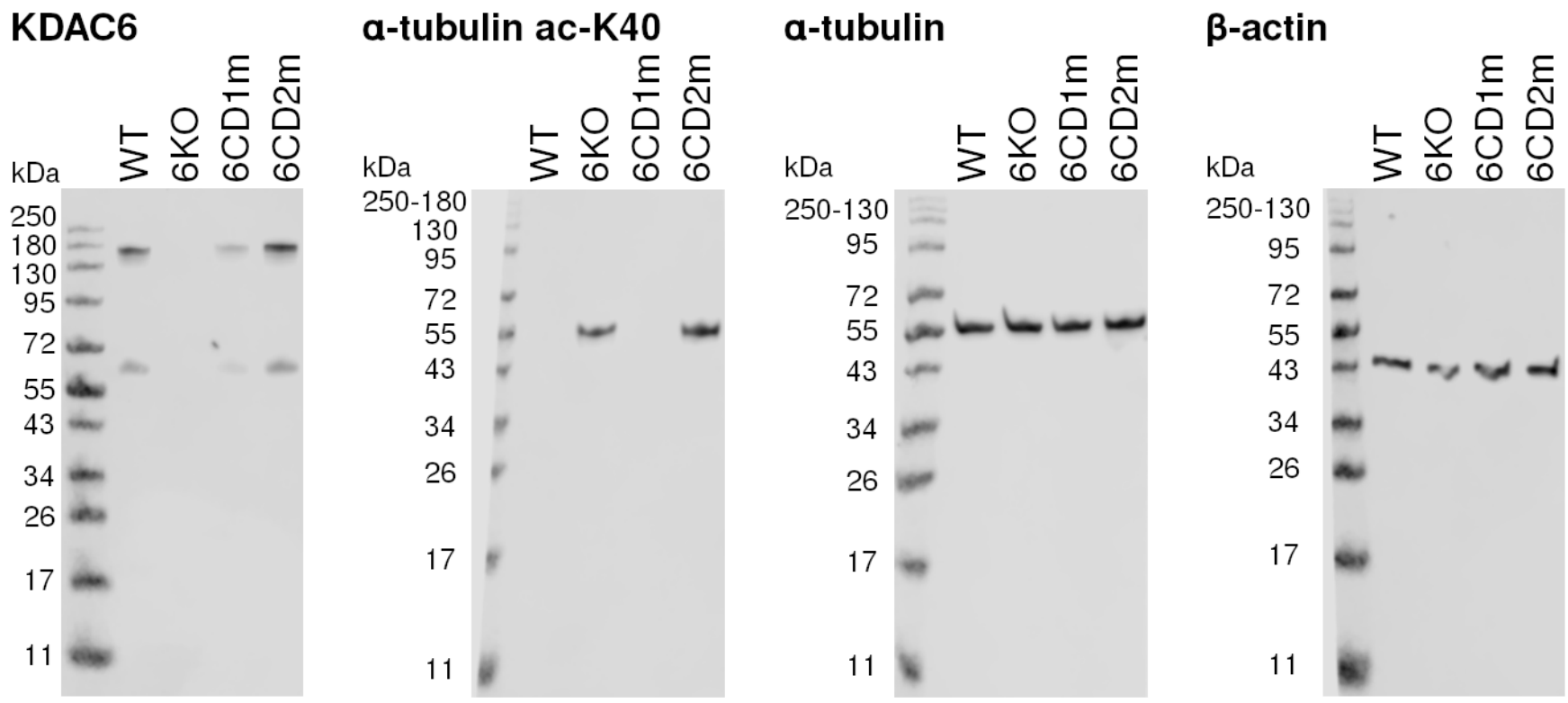

Figure S1. Full immunoblots from figure 1. Full panels for all cropped blots in figure 1. The smaller band in the KDAC6 blot was consistent in proportional intensity relative to the larger band in several replicate blots from multiple lysate samples.

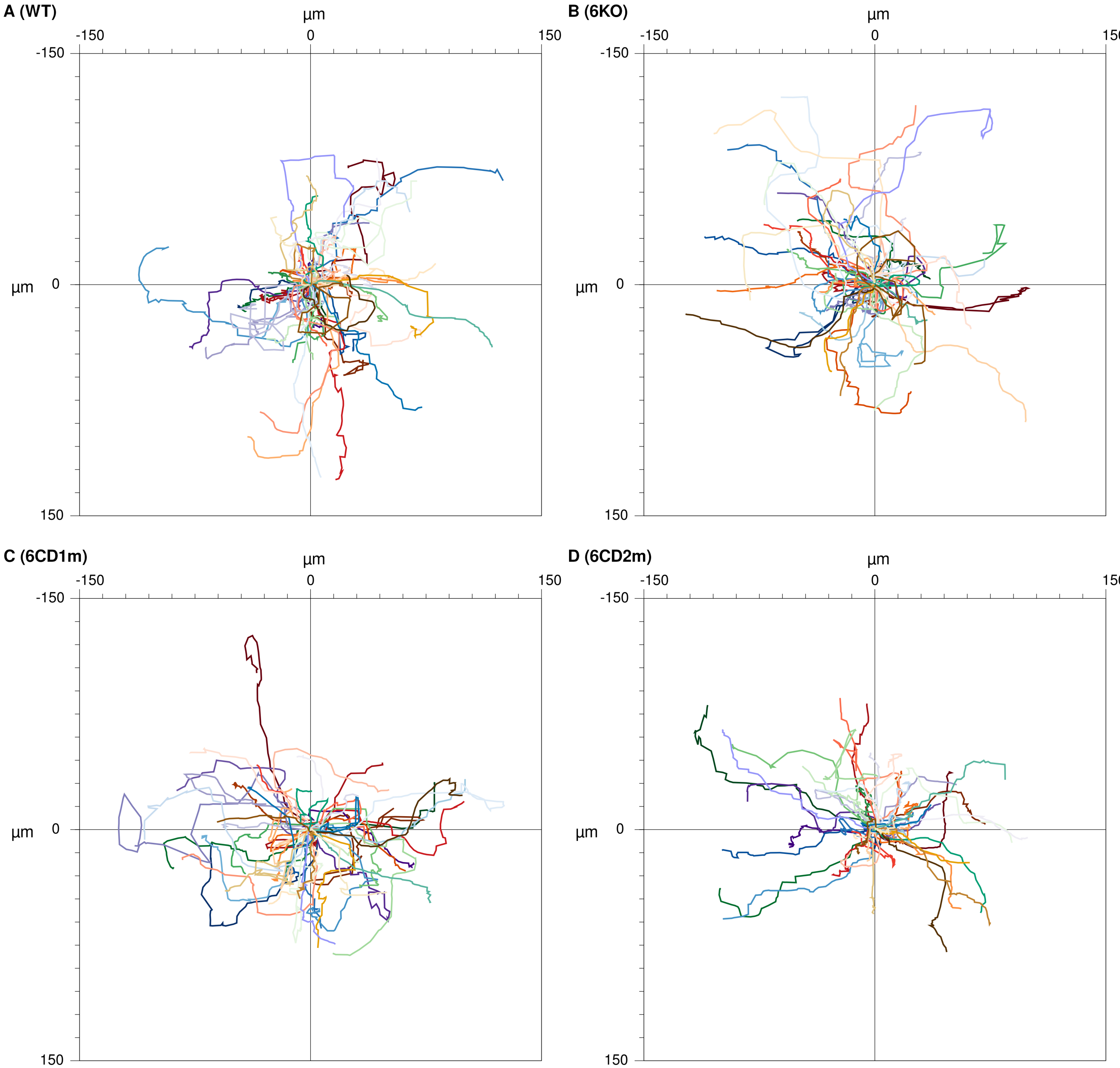

Figure S2. KDAC6 variants demonstrate distinct single-cell migration patterns. Time-lapse images were collected for low density cells of (A) wild-type, (B) 6KO, (C) 6CD1m, and (D) 6CD2m. The MTrackJ plugin for ImageJ was used to track the movement of individual, isolated cells for 4 hours. The lines correspond to 50 representative tracks for each cell line.

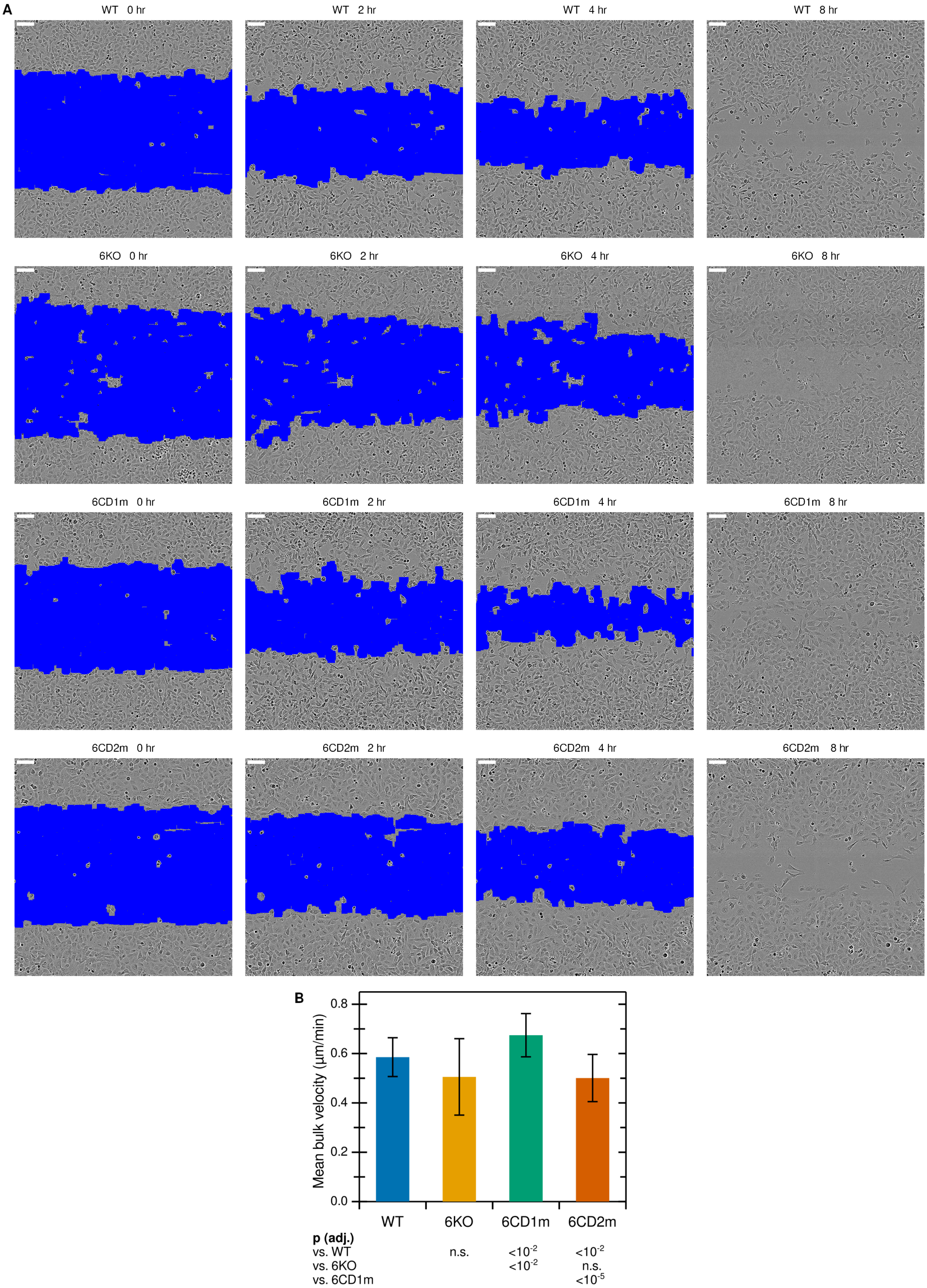

Figure S3. KDAC6 CD1 and CD2 inactivation cause opposite changes to bulk cell migration. (A) Representative images of scratch assay for each cell line to measure bulk migration rate. The blue area represents the scratched area containing no cells at 0, 2, and 4 hours. Images at 8 hours illustrate progress toward closure at a late time point. Scale bars represent 100 µm. All image intensities were normalized to a common range with cropping of less than 1% of the total signal. (B) Mean bulk velocity for each cell line ($n \geq 16$ for each cell line). Images from scratch assay were analyzed to calculate the velocity of bulk cell movement using the change in position of the cell front represented by the edges of the blue area for each cell line during the first 4 hours post-scratch. Error bars represent standard deviation, and p values were determined by Mann-Whitney U tests.

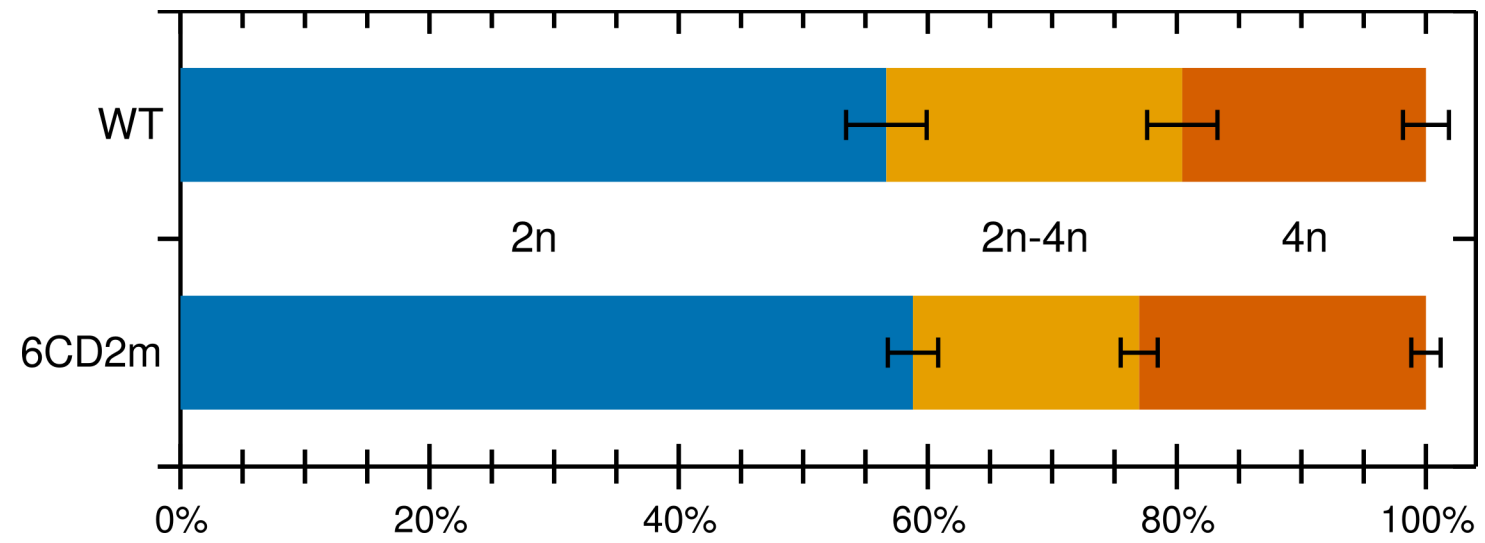

Figure S4. DNA content is not significantly changed in 6CD2m cells. The percentage of the total cell population having 2n, 4n, or an intermediate amount of DNA content in wild-type and 6CD2m cells as measured by pooled flow cytometry data (≥ 6 independent experiments for each cell line; n ≥ 930,000 total events for each cell line). DNA content is labeled for each bar section. Error bars represent standard error of the mean of the independent experiments. The proportion of cells in each group is not statistically different (pairwise Mann-Whitney U tests, $p > 0.05$).

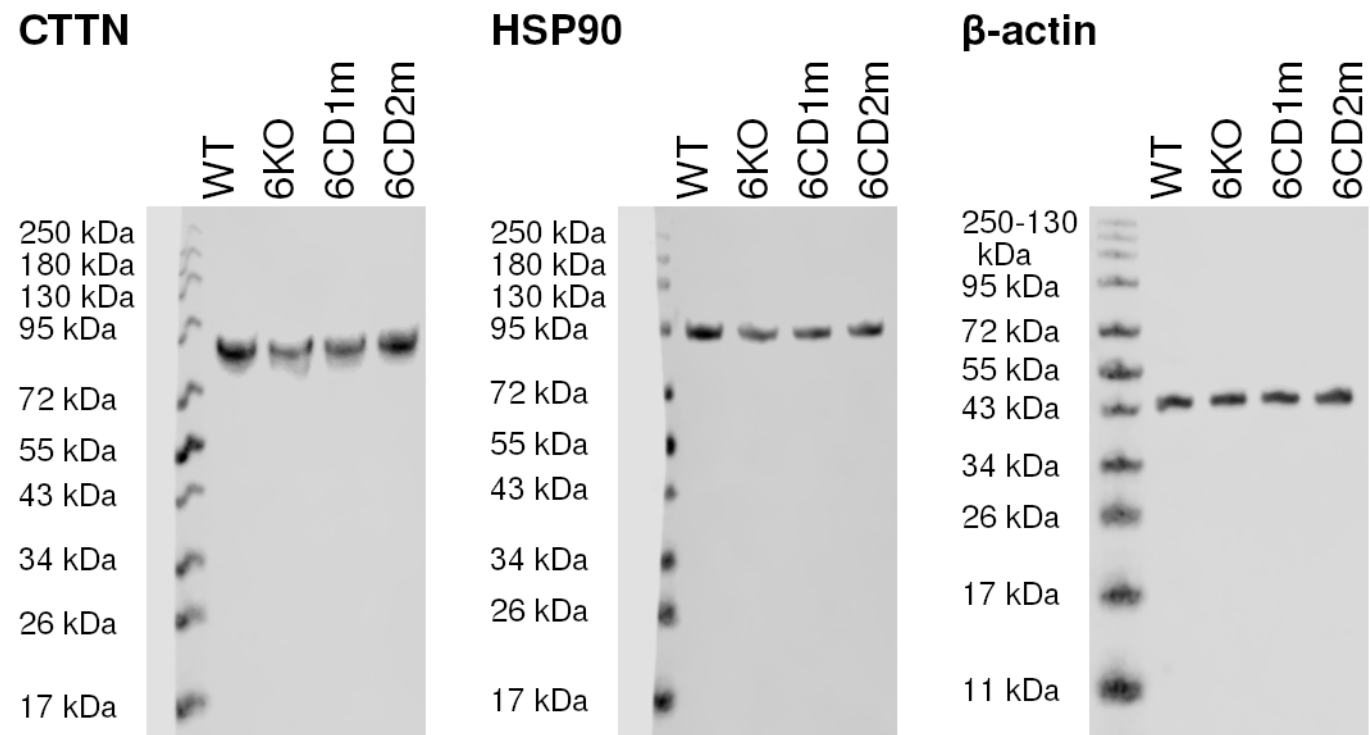

Figure S5. Full immunoblots from figure 7. Full panels for all cropped blots in figure 7.
